# Sensing of Ebinur Lake virus by distinct pattern recognition receptors dictates cell-type specific innate immunity and pathogenesis

**DOI:** 10.1128/jvi.00750-25

**Published:** 2025-09-15

**Authors:** Jia-Peng Zou, Su-Yun Wang, Han Xia, Zhi-Sheng Xu, Wei-Wei Luo, Yan-Yi Wang

**Affiliations:** 1 State Key Laboratory of Virology and Biosafety, Chinese Academy of Sciences, Wuhan Institute of Virology 74614 , Wuhan, China; 2 University of Chinese Academy of Sciences 74519 , Beijing, China; University Medical Center Freiburg, Freiburg, Germany

**Keywords:** pattern recognition receptors, pathogen-associated molecular patterns, innate immunity, host-pathogen interactions, orthobunyavirus

## Abstract

Ebinur Lake virus (EBIV) is a recently identified orthobunyavirus with broad host range and zoonotic potential, posing a public health risk. However, the mechanisms underlying EBIV pathogenesis and host innate immune responses remain unclear. Here, we investigated the pattern recognition receptors (PRRs) responsible for sensing EBIV infection and subsequent pathogenesis. EBIV infects diverse cell types and exhibits broad tissue tropism *in vivo. In vitro*, RIG-I was essential for type I interferon (IFN-I) and inflammatory responses in HEK293 and A549 cells. In contrast, both RIG-I and MDA5 contributed to IFN-I induction in Huh-7 and HCT116 cells, correlating with the specific accumulation of viral dsRNA intermediates in these cell types. Both RIG-I and MDA5 preferentially recognize RNA derived from the viral S segment; however, they have different abilities in sensing incoming viral genomic RNA bearing a 5'-phosphate motif and the replication intermediates. *In vivo*, RIG-I deficiency severely impairs host defense, while MDA5 deficiency has a more restricted effect in the spleen and liver. In addition to RIG-I and MDA5, TLR7, which is predominantly expressed in dendritic cells, also plays a crucial role for host defense by mediating systemic inflammatory cytokine production without significantly impacting IFN-I response. Our findings suggest that multiple innate sensing receptors, including RIG-I, MDA5, and TLR7, are differentially involved in host defense against EBIV by mediating IFN-I and inflammatory responses, respectively, in a cell-specific manner.

**IMPORTANCE:**

This study elucidates the complex mechanisms by which host RIG-I, MDA5, and TLR7 sense the emerging EBIV and trigger cell-specific immune responses. These findings not only clarify crucial aspects of EBIV-host interactions, particularly the differential sensing of viral RNA by distinct PRRs, but also underscore how this differential sensing dictates cell-specific innate immune activation (IFN-I vs. inflammatory responses) and viral pathogenesis, providing critical insights for understanding and combating EBIV and related emerging bunyaviruses.

## INTRODUCTION

The emergence of pathogenic viruses poses a critical challenge to public health (1). Ebinur Lake virus (EBIV) is a recently identified member of the Bunyavirales order (2), a group of viruses known for their segmented RNA genomes and their potential to cause severe diseases in humans and animals (3). EBIV was first isolated from Culex mosquitoes in the Ebinur Lake region of Northwest China (2, 4). Recent studies have demonstrated that EBIV can efficiently replicate and cause cytopathic effects in a wide variety of cells derived from mosquitoes, rodents, monkeys, and humans (4, 5). Moreover, EBIV infection at low dose (one plaque-forming unit [PFU]) causes necrosis and hemorrhage of multiple organs, including the liver, lung, intestine, and kidney, and death in wild-type BALB/c mice (4, 5). Serological surveys among local residents have shown positive antibody response against EBIV, indicating its potential risk for public health (4). Despite these findings, the mechanisms of viral pathogenesis and host innate immune response to EBIV infection remain poorly understood.

Viral infection is sensed by pattern recognition receptors (PRRs) of the host innate immune system, which recognize the conserved viral structural components called pathogen-associated molecular patterns (PAMPs). Sensing of PAMPs by PRRs triggers innate immune signaling pathways that lead to induction of downstream antiviral genes, including type I interferons (IFN-I) and proinflammatory cytokine genes (6). Among the known PRRs, several are specialized in recognizing viral RNA, including members of the Toll-like receptor (TLR), RIG-I-like receptor (RLR) and NOD-like receptor (NLR) families, and double-stranded RNA activated protein kinase R (PKR) (6). TLRs are receptors located on cell membranes or within endosomal compartments, among which TLR3 recognizes dsRNA, a replication intermediate for many RNA viruses (7). Endosomal TLR7 and TLR8 detect single-stranded RNA (ssRNA) and are involved in recognizing viral RNA derived from various viruses, such as influenza virus and SARS-CoV-2 (7). Recent studies show that some NLRs, such as NLRP6 and NLRP9b, in complex with specific DEAH-box RNA helicases, can sense viral dsRNA and activate type I IFN- and/or inflammasome-dependent antiviral responses (8, 9). In addition, PKR also recognizes dsRNA, leading to the phosphorylation and inactivation of eIF2α to inhibit protein synthesis in infected cells (10).

RLRs, including RIG-I (retinoic acid-inducible gene I) and MDA5 (melanoma differentiation-associated protein 5), are sensors that detect viral RNA in the cytosol, where many RNA viruses replicate (11). These receptors share a homologous structure, including a central helicase domain and a carboxy-terminal domain (CTD) that is responsible for RNA recognition. RIG-I and MDA5 additionally have two N-terminal caspase activation and recruitment domains (CARDs) that are essential for signal transduction (12, 13). Although structurally homologous, RIG-I and MDA5 discriminate among different ligands to trigger innate immune response. RIG-I recognizes short dsRNA and ssRNA with a 5′-triphosphate end, which is common in viral, but not, host RNAs (14, 15). In the case of DNA virus infection, poly(dA:dT) fragments of viral genomic DNA can be transcribed by RNA polymerase III, and the resulting RNA is detected by RIG-I to trigger innate immune response (16, 17). MDA5 preferentially detects long irregular dsRNA that presumably exists in a complex higher-order configuration (18, 19). Accumulating evidence demonstrates that RIG-I and MDA5 can act distinctly or cooperatively to sense infection of certain viruses. Negative-strand (ns) RNA viruses of the *Paramyxoviridae*, *Orthomyxoviridae*, *Rhabdoviridae*, *Bunyaviridae*, and *Filoviridae* families are mainly sensed by RIG-I, whereas positive-strand RNA viruses of the *Picornaviridae* family are sensed by MDA5 (18, 20, 21). The sensing of *Reoviridae* and *Flaviviridae* family members is mediated by both RIG-I and MDA5, highlighting the collaboration of the two receptors in antiviral immunity (21, 22). Upon binding to viral RNA, RIG-I or MDA5 undergoes conformational change, leading to its recruitment to the CARD domain-containing adaptor VISA (also called MAVS, IPS-1, and Cardif), located either at the mitochondria or the peroxisomes (23–27). This triggers signaling that results in translocation of the transcription factors IRF3 and NF-κB into the nucleus, initiating transcriptional induction of downstream antiviral effectors including type I IFNs and proinflammatory cytokines. Secreted type I IFNs bind to their receptors, activating the JAK/STAT signaling pathways to induce the expression of numerous IFN-stimulated genes (ISGs) (28). The ISGs encode various proteins that inhibit viral replication, induce cell death, boost antigen presentation, and modulate the immune response (28).

In this study, we demonstrate that EBIV infects a broad range of human and murine cell types. We show that RIG-I, MDA5, and TLR7 are differentially involved in sensing of EBIV in different cell types, which are determined by exposure to differential PAMPs during EBIV infection, including the viral genomic RNA containing 5' triphosphate or bisphosphate motif and dsRNA produced during replication. Experiments with gene knockout mice reveal that RIG-I, MDA5, and TLR7 differentially contribute to host innate immune and inflammatory responses in responding to EBIV. Our findings elucidate the precise mechanisms of innate immune sensing and signaling of EBIV, which is critical to understand EBIV-host interactions.

## RESULTS

### Tropism of EBIV in cells and tissues

**Fig 1:**
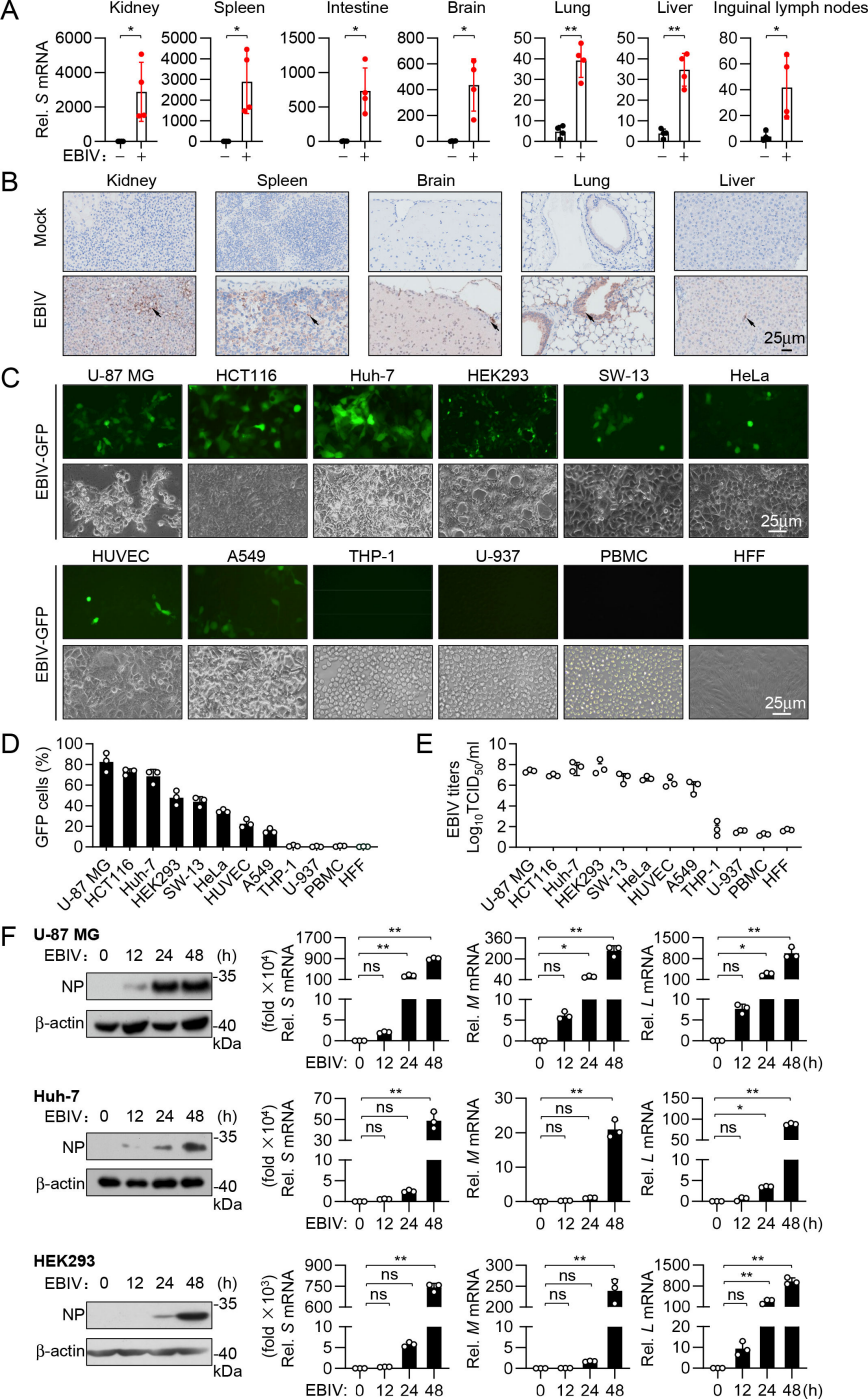
Tropisms of EBIV in cells and tissues. (**A**) RT-qPCR analysis of EBIV S RNA in the indicated tissues from WT mice (*n* = 4) infected i.p. with EBIV (1 × 10^3 PFU) for 48 hours. (**B**) IHC staining of EBIV N protein (brown dots, arrows) in the tissues from mice treated as in (**A**). Scale bar, 25 µm. (**C and D**) EBIV infects a wide range of human cell lines. The indicated cell lines were infected with EBIV-GFP (MOI = 1) for 48 hours, followed by imaging by fluorescence microscope (**C**) or quantified by flow cytometry (**D**). Scale bar, 25 µm. (**E**) The indicated cell lines were infected with EBIV (MOI = 1) for 48 hours, and the production of EBIV progeny viruses in supernatants was measured by TCID_50_ assay. (**F**) U-87 MG, Huh-7, and HEK293 cells were infected with EBIV (MOI = 1) for the indicated times; levels of N protein and viral RNA were measured by immunoblotting and RT-qPCR, respectively. Data are shown as mean ± SD (*n* = 4 mice A; *n* = 3 independent cell samples, **C–F**). Statistical significance was determined by unpaired two-tailed Student’s *t*-test (**A**) or one-way ANOVA (**F**). *P < 0.05; **P < 0.01. ns, not significant.

To investigate the tissue tropism of EBIV *in vivo*, we systematically collected various tissues (kidney, spleen, intestine, brain, lung, liver, and lymph nodes) from C57BL/6 mice infected with EBIV and assessed viral presence using quantitative PCR (qPCR) for viral RNA and immunohistochemistry (IHC) with an anti-NP antibody. The results indicated that EBIV infected and replicated in a broad range of tissues *in vivo* (Fig. 1A and B). To complement tissue-level analysis, we examined EBIV infectivity in primary murine cells. The results indicated that mouse lung fibroblasts (MLFs), mouse embryonic fibroblasts (MEFs), and mouse bone marrow-derived dendritic cells (BMDCs) were susceptible to EBIV infection, while mouse bone marrow-derived macrophages (BMDMs) were largely non-permissive (Fig. S1A through C). Next, we evaluated the infectivity of EBIV in human cells. We first evaluated the infection and replication of GFP-tagged EBIV in various human cell lines by monitoring GFP expression levels and the percentage of GFP-positive cells. Our results indicated that seven human cell lines, including brain glioblastoma U-87 MG, liver carcinoma Huh-7, embryonic kidney HEK293, cervical epithelial cancer HeLa, adrenal gland and cortex epithelial SW-13, colon cancer HCT116, and lung epithelial A549 cells, were susceptible to EBIV infection to varied degrees, whereas minimal infection was observed in human monocytic THP-1 and U937 cells (Fig. 1C and D). Moreover, cytopathic effects were observed in the susceptible cells 48–60 hours post-infection with EBIV (Fig. S1D). Productive replication in these cell lines was confirmed by measuring progeny virus titers (Fig. 1E) and detecting viral S, M, and L segment RNAs and N protein expression (Fig. 1F). We also further tested the infection tropism of EBIV in primary human cells. We found that human umbilical vein endothelial cells (HUVECs) were permissive to EBIV infection, whereas human foreskin fibroblasts (HFFs) and peripheral blood mononuclear cells (PBMCs) were largely non-permissive under our experimental conditions (Fig. 1E; Fig. S1D). Together, these results suggest that EBIV exhibits a broad tropism, infecting multiple tissues *in vivo* as well as diverse human and murine cell lines and specific primary cell types, including endothelial cells and dendritic cells.

### EBIV triggers innate immune response in infected cells

**Fig 2:**
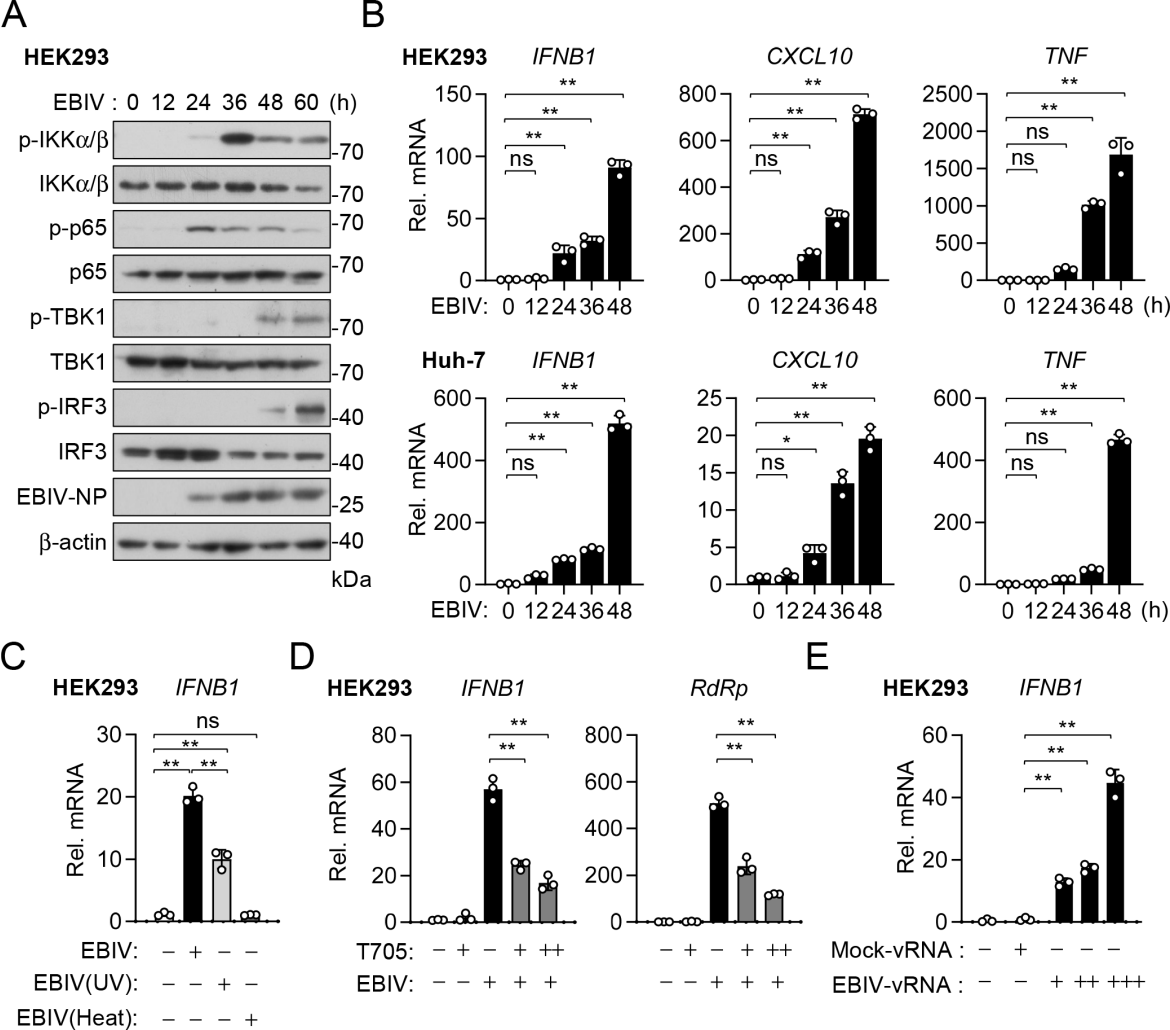
EBIV activates innate immune responses in infected cells. (**A**) Immunoblot analysis of the indicated proteins in HEK293 cells uninfected and infected with EBIV (MOI = 1) for the indicated times. (**B**) RT-qPCR analysis of *IFNB1*, *CXCL10*, and *TNF* mRNAs in HEK293 and Huh-7 cells uninfected and infected with EBIV (MOI = 1) for the indicated times. (**C**) RT-qPCR analysis of *IFNB1* mRNA in HEK293 cells infected with untreated, UV-treated (248 nm UV for 10 min), or heat-treated EBIV (65℃ for 20 minutes) for 24 hours. (**D**) RT-qPCR analysis of the indicated mRNA in HEK293 cells untreated or treated with T705 (50, 100 nM) for 12 hours and then infected with EBIV (MOI = 1) for 24 hours. (**E**) vRNA was isolated from supernatants of Vero cells infected with EBIV (MOI = 1) for 24 hours. RT-qPCR analysis of *IFNB1* mRNA in HEK293 cells transfected with vRNA (0.5, 1, 2 µg/mL) for 12 hours. Data are shown as mean ± SD (*n* = 3 independent cell samples, **B–E**). Statistical significance was determined by one-way ANOVA (**B–E**). *P < 0.05; **P < 0.01. ns, not significant.

Sensing of viral infection by PRRs leads to induction of numerous antiviral genes and innate immune response. To determine whether EBIV infection effectively activates cellular innate immune response, we examined the activation of PRR-triggered signaling pathways in HEK293 cells, which are highly susceptible to EBIV infection. We found that phosphorylation of IKKα/β, p65, TBK1, and IRF3, which are hallmarks of activation of innate immune signaling, was markedly induced following EBIV infection in HEK293 cells (Fig. 2A). Notably, phosphorylation of IKKα/β and p65, which are essential for activation of the NF-κB signaling pathway, was initiated at 24 hours after EBIV infection, whereas phosphorylation of TBK1 and IRF3, which drive the expression of ISRE-containing genes, was only detectable at 48 hours after EBIV infection (Fig. 2A). Consistently, EBIV infection induced the transcription of downstream *IFNB1*, *CXCL10*, and *TNF* genes in HEK293 and Huh-7 cells at 24–48 hours after infection (Fig. 2B). To investigate the contribution of viral replication to this response, we treated EBIV with UV light or heat prior to infection. UV treatment, which primarily damages viral nucleic acids, significantly reduced (by ~60%) the virus’s ability to induce *IFNB1* transcription, while heat treatment, which denatures proteins and inhibits entry/replication, completely abolished induction (Fig. 2C). To confirm this, we examined the effects of viral RNA polymerase (RdRp) inhibitor Favipiravir (T705) on EBIV-induced innate immune signaling. The results indicated that pretreatment of cells with Favipiravir inhibited EBIV replication in a dose-dependent manner and correspondingly suppressed EBIV-induced *IFNB1* mRNA expression (Fig. 2D). These results suggest that viral replication is required for efficiently initiating innate immune response. We next assessed whether the incoming viral genome itself possesses immunostimulatory activity. Indeed, transfection of purified viral genomic RNA (vRNA) into cells induced *IFNB1* transcription in a dose-dependent manner compared to control RNA (Fig. 2E). Collectively, these results demonstrate that EBIV infection activates canonical innate immune signaling pathways, and while incoming vRNA can trigger a baseline response, active viral replication is necessary for the full induction of antiviral genes.

### RLRs are essential for antiviral innate immune response to EBIV *in vivo*

**Fig 3:**
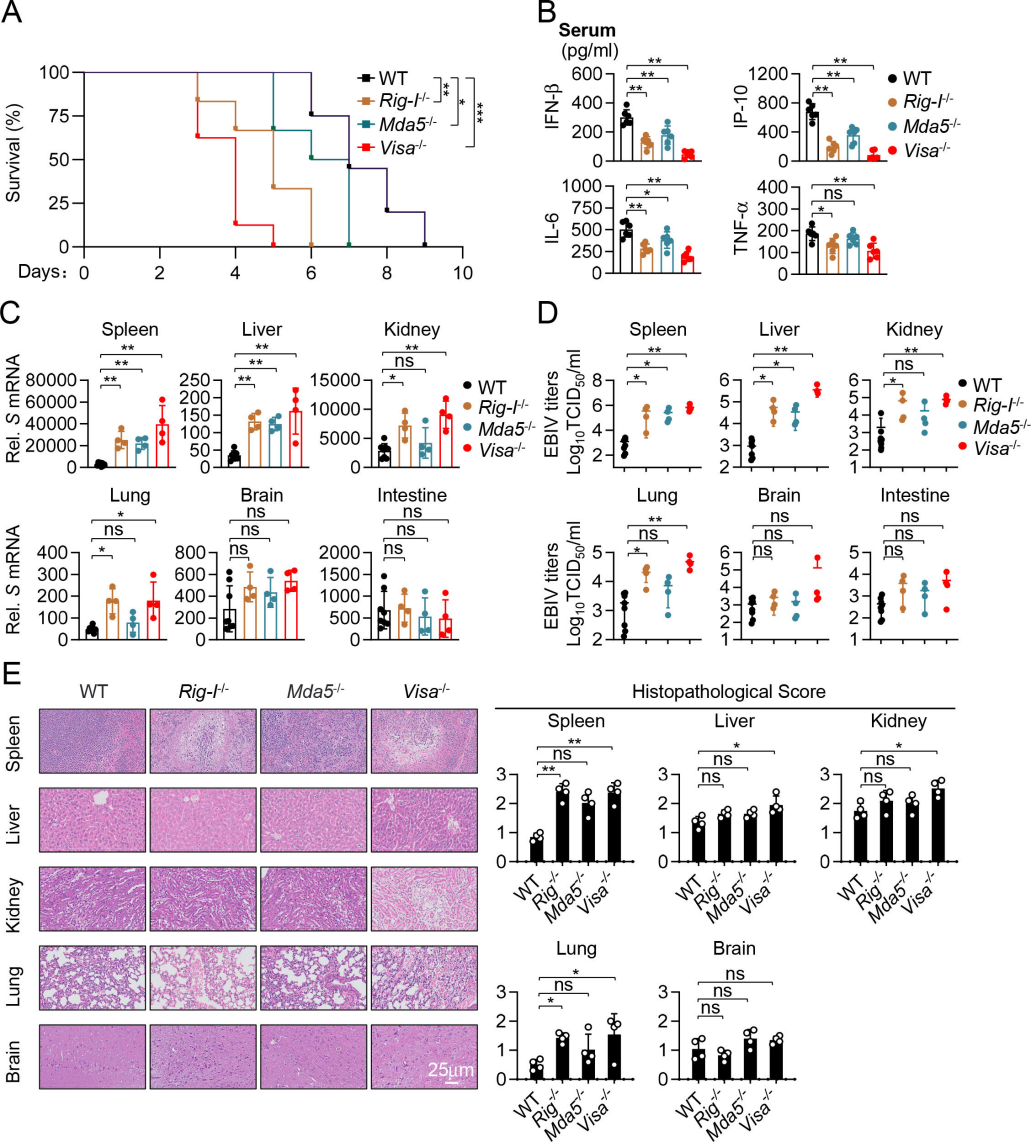
RLR-VISA signaling is essential for antiviral innate immune response to EBIV *in vivo*. (**A**) Survival of WT (*n* = 20), *Rig-I*^-/-^ (*n* = 6), *Mda5*^-/-^ (*n* = 6), and *Visa*^-/-^ (*n* = 8) mice after i.p. injection with EBIV (1 × 10^3^ PFU per mouse). (**B**) ELISA analysis of the indicated cytokines in sera of the indicated mice (*n* = 6) infected for 48 hours by i.p. injection of EBIV (1 × 10^3^ PFU per mouse). (**C–E**) WT (*n* = 8), *Rig-I*^-/-^ (*n* = 4), *Mda5*^-/-^ (*n* = 4), and *Visa*^-/-^ (*n* = 4) mice were infected for 48 hours by i.p. injection of EBIV (1 × 10^3^ PFU per mouse). The indicated tissues were collocated for RT-qPCR analysis (**C**), EBIV titer analysis (**D**), and H&E staining with semi-quantitative scoring of histopathology (**E**). Scale bar, 25 µm. Data are shown as mean ± SD (**B–E**). Statistical significance was determined by the log-rank Mantel-Cox test (**A**), one-way ANOVA (**B**), or unpaired two-tailed Student’s *t*-test (**C–E**). *P < 0.05; **P < 0.01. ns, not significant.

We next investigated the *in vivo* roles of the RLR sensors RIG-I and MDA5, and their essential adaptor VISA, in host defense against EBIV. We infected 8-week-old WT, RIG-I-deficient (*Rig-I*^−/−^), MDA5-deficient (*Mda5*^−/−^), and VISA-deficient (*Visa*^−/−^) mice with a lethal dose of EBIV intraperitoneally (i.p.) and monitored their survival. Compared to wild-type mice, *Rig-I*^−/−^ mice were highly susceptible to EBIV, showing markedly accelerated mortality compared to WT mice infected with EBIV. *Mda5*^−/−^ mice displayed increased susceptibility compared to WT mice but were less susceptible compared to *Rig-I*^−/−^ mice. In these experiments, *Visa*^−/−^ mice exhibited the highest susceptibility and lethality to EBIV infection among all the examined groups (Fig. 3A). Consistently, knockout of RIG-I significantly reduced EBIV-induced secretion of serum IFN-β, IP-10, IL-6, and TNF-α compared to wild-type mice (Fig. 3B). In contrast, knockout of MDA5 moderately reduced EBIV-induced secretion of IFN-β, IP-10, and IL-6, but did not have marked effects on serum TNF-αlevel (Fig. 3B). In these experiments, knockout of VISA reduced EBIV-induced secretion of serum IFN-β, IP-10, IL-6, and TNF-α to lower levels than knockout of either RIG-I or MDA5 (Fig. 3B). Consistent with impaired antiviral responses, viral replication and production, assessed by S segment RNA levels and measuring progeny virus titers, were significantly elevated in multiple tissues (including spleen, liver, kidney, and lung) of *Rig-I*^−/−^ and *Visa*^−/−^ mice compared to WT controls (Fig. 3C and D). A significant increase in viral loads in *Mda5*^−/−^ mice compared to wild-type mice was only detected in the spleen and liver, but not in other examined tissues (Fig. 3C and D). Correspondingly, histopathological analysis revealed more severe inflammatory infiltrates and tissue damages in the spleen, liver, kidney, and lung of *Visa*^−/−^ mice compared to WT mice, correlating with the higher viral burdens in these tissues (Fig. 3E). RIG-I deficiency resulted in significantly increased pathology scores specifically in the spleen and lung, but not in the liver, kidney, or brain compared to WT mice (Fig. 3E). Notably, MDA5 deficiency led to significantly elevated pathology scores only in the spleen (Fig. 3E). The observation that VISA deficiency exacerbates pathology in the liver and kidney, while deficiency of either RIG-I or MDA5 does not, suggests potential complementary or redundant roles for RIG-I and MDA5 in limiting immunopathology specifically within these organs. Collectively, these results demonstrate that VISA-dependent signaling is critical for controlling EBIV replication and limiting immunopathology *in vivo*, with RIG-I playing a major role, while MDA5 appears more restricted in certain tissues like the spleen and liver.

### TLR7 is important for inflammatory response induced by EBIV in DCs

**Fig 4:**
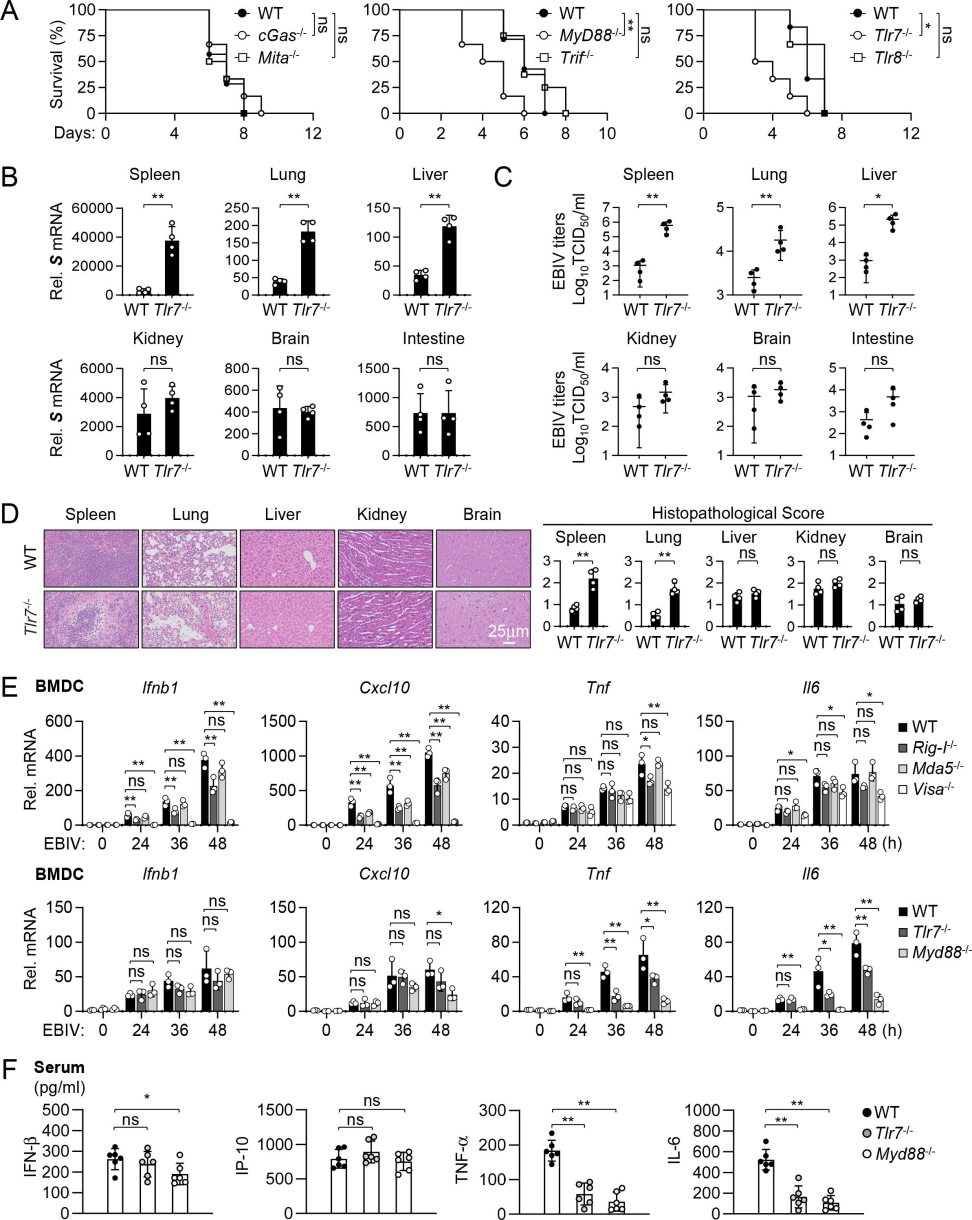
TLR7-MyD88 axis is important for inflammatory response induced by EBIV in mice. (**A**) Survival of the indicated mice after i.p. injection with EBIV (1 × 10^3^ PFU per mouse). Left panel: WT (*n* = 7), *cGas*^-/-^ (*n* = 6), and *Mita*^-/-^ (*n* = 6); middle panel: WT (*n* = 14), *Myd88*^-/-^ (*n* = 6), and *Trif*^-/-^ (*n* = 8); right panel: WT (*n* = 6), *Tlr7*^-/-^ (*n* = 6), and *Tlr8*^-/-^ (*n* = 6). (**B–D**) WT (*n* = 4) and *Tlr7*^-/-^ (*n* = 4) mice were infected for 48 hours by i.p. injection of EBIV (1 × 10^3^ PFU per mouse). The indicated tissues were collocated for RT-qPCR analysis (B), EBIV titer analysis (**C**), and H&E staining with semi-quantitative scoring of histopathology (**D**). Scale bar, 25 µm. (**E**) RT-qPCR analysis of the indicated genes in BMDCs from WT, *Rig-i*^-/-^, *Mda5*^-/-^, *Visa*^-/-^, *Tlr7*^-/-^, and *Myd88*^-/-^ mice after EBIV infection (MOI = 1) for the indicated times. (**F**) ELISA analysis of the indicated cytokines in sera of WT, *Tlr7*^-/-^, and *Myd88*^-/-^ mice infected for 48 hours by i.p. injection of EBIV (1 × 10^3^ PFU per mouse). Data are shown as mean ± SD (**B–F**). Statistical significance was determined by the log-rank Mantel-Cox test (**A**), unpaired two-tailed Student’s *t*-test (**B–D**), and one-way ANOVA (E–F). *P < 0.05; **P < 0.01. ns, not significant.

The above *in vivo* experiments indicated that deficiency of the RLR-VISA axis did not completely abolish EBIV-induced production of innate immune and inflammatory cytokines (Fig. 3B). This led us to hypothesize that additional innate immune sensors are involved in host defense to EBIV. Recent studies suggest that certain RNA viruses induce mitochondrial stress, leading to release of mtDNA into the cytosol to trigger innate immune and inflammatory response via the cGAS-MITA/STING axis (29–31). However, infecting *cGas*^−/−^ and *Mita*^−/−^ mice with lethal doses of EBIV revealed comparable lethality to WT mice (Fig. 4A), suggesting that the cGAS-MITA axis is dispensable for host defense against EBIV.

We next considered endosomal TLRs known to recognize viral RNA, namely TLR3, TLR7, and TLR8 (7). Since these TLRs signal through the adaptor proteins MyD88 and/or TRIF (7), we examined the roles of these adaptors in host defense against EBIV *in vivo*. MyD88-deficient mice exhibited significantly increased susceptibility to EBIV compared to WT mice, whereas TRIF-deficient mice showed comparable lethality (Fig. 4A). Given that TLR7/8 signals through MyD88, while TLR3 signals through TRIF (7), we therefore hypothesized that TLR7 and/or TLR8, but not TLR3, were involved in antiviral response in EBIV-infected mice. Subsequent experiments showed that TLR7-deficient mice were significantly more susceptible to EBIV infection, while TLR8-deficient mice exhibited survival similar to WT mice (Fig. 4A). These results suggest that TLR7-MyD88 signaling plays a role in host defense against EBIV *in vivo*. Consistent with increased susceptibility, *Tlr7*^−/−^ mice displayed significantly higher viral burden compared to WT mice at 48 hours post-infection, specifically in the spleen, lung, and liver, as measured by both S segment RNA levels (Fig. 4B) and infectious virus titers (TCID50) (Fig. 4C). In contrast, viral loads and titers in the kidney, brain, and intestine were not significantly different between *Tlr7*^−/−^ and WT mice (Fig. 4B and C). Correspondingly, histopathological analysis revealed significantly higher pathology scores in the spleen and lung of *Tlr7*^−/−^ mice compared to WT controls, while scores in the liver, kidney, and brain were comparable between the genotypes (Fig. 4D). These findings suggest that TLR7 plays a tissue-specific role in controlling EBIV replication and limiting associated pathology.

Since TLR7 is predominantly expressed in dendritic cells (DCs) (7) and EBIV efficiently infects BMDCs (Fig. S1C), we investigated TLR7-MyD88 signaling in EBIV-infected BMDCs. qPCR revealed that EBIV-induced transcription of *Ifnb1* and *Cxcl10* was impaired in BMDCs lacking RIG-I or VISA, but not MDA5 (Fig. 4E). Conversely, RIG-I or VISA deletion had minimal impact on the induction of inflammatory genes *Tnf* and *Il6* in BMDCs (Fig. 4E). In contrast, knockout of TLR7 or MyD88 did not affect expression of *Ifnb1* and *Cxcl10* but significantly inhibited expression of *Tnf* and *Il6* induced by EBIV (Fig. 4E). This pattern was mirrored in systemic cytokine production in mice. Serum levels of the inflammatory cytokines TNF-α and IL-6 were markedly reduced in *Tlr7*^−/−^ or *Myd88*^−/−^ mice compared to WT mice post-infection (Fig. 4F). Importantly, knockout of TLR7 or MyD88 did not significantly affect EBIV-induced serum levels of IFN-β or IP-10 (Fig. 4F). Together, these findings suggest that the TLR7-MyD88 axis plays a crucial role specifically in the inflammatory cytokine response to EBIV, thereby complementing the primary antiviral IFN/ISG response driven by the RLR-VISA pathways.

### RIG-I is essential for innate immune response to EBIV in HEK293 and A549 cells

**Fig 5:**
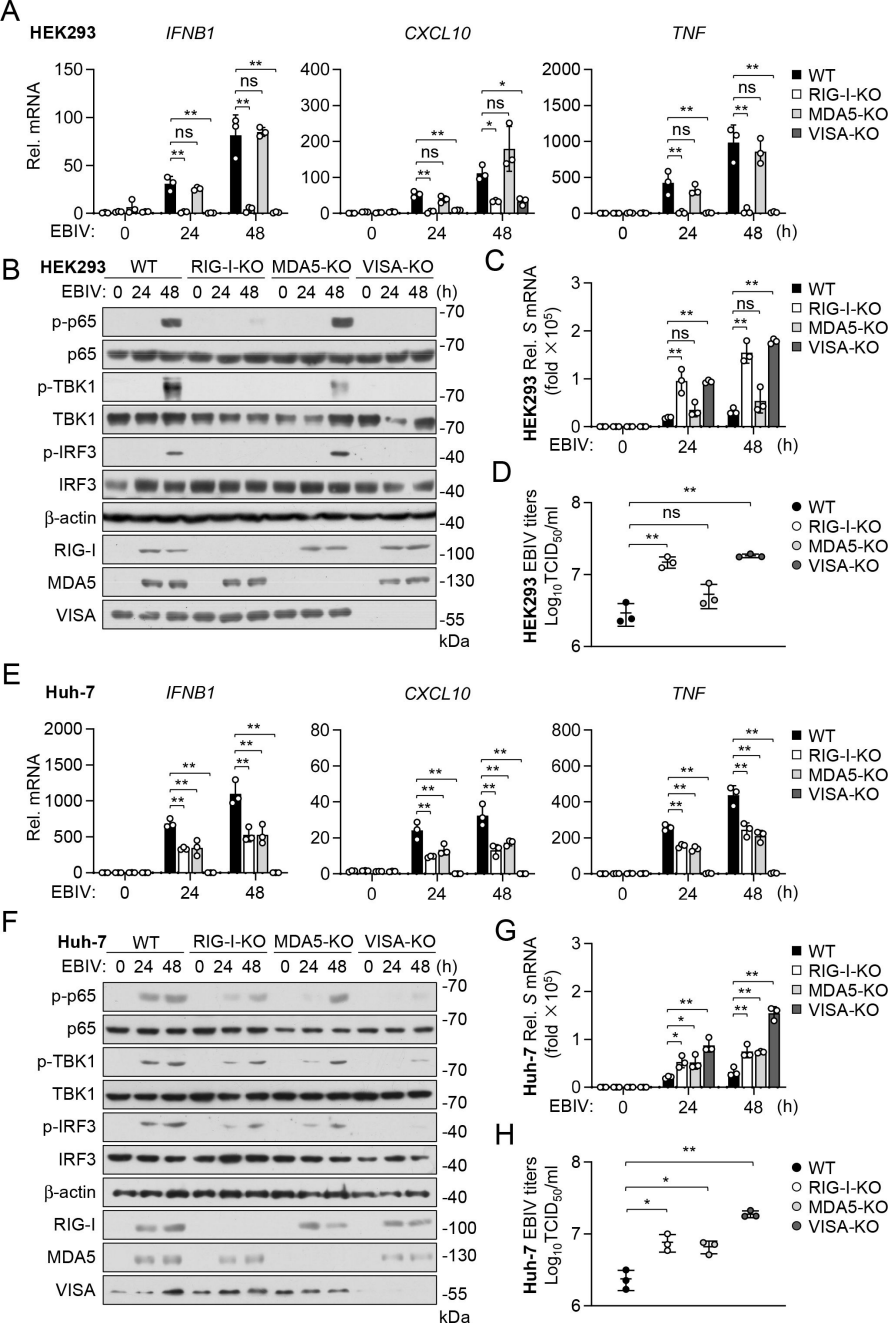
Differential roles of RIG-I and MDA5 in innate immune sensing of EBIV in distinct cell types. (**A–D**) Wild-type (WT), RIG-I-deficient (RIG-I-KO), MDA5-deficient (MDA5-KO), and VISA-deficient (VISA-KO) HEK293 cells were uninfected and infected with EBIV (A, C–D, MOI = 1; B, MOI = 5) for the indicated times. The mRNA levels of the indicated genes were measured by RT-qPCR (**A, C**). The indicated proteins were detected by immunoblot analysis (**B**). Production of EBIV progeny viruses in supernatants was measured by TCID_50_ assay (**D**). (**E–H**) WT, RIG-I-, MDA5-, and VISA-deficient Huh-7 cells were uninfected and infected with EBIV (E, G–H, MOI = 1; F, MOI = 5) for the indicated times. The mRNA levels of the indicated genes were measured by RT-qPCR (**E, G**). The indicated proteins were detected by immunoblot analysis (**F**). Production of EBIV progeny viruses in supernatants was measured by TCID_50_ assay (**H**). Data are shown as mean ± SD (*n* = 3 independent cell samples, **A, C–D, E, G–H**). Statistical significance was determined by one-way ANOVA. *P < 0.05; **P < 0.01. ns, not significant.

Having established the critical roles of RLR-VISA and TLR7-MyD88 signaling *in vivo*, we next investigated the specific contributions of the RLR sensors RIG-I and MDA5 in mediating the innate immune response within different human cell lines *in vitro*. We used CRISPR-Cas9 to knock out RIG-I, MDA5, or VISA in HEK293 cells and assessed their roles in innate immune response to EBIV. RT-qPCR results showed that knockout of RIG-I or VISA reduced the induction of *IFNB1*, *CXCL10*, and *TNF* genes induced by EBIV in HEK293 cells, whereas knockout of MDA5 had no marked effects (Fig. 5A). Additionally, EBIV-induced phosphorylation of TBK1, IRF3, and p65 was eliminated in RIG-I- and VISA-deficient HEK293 cells, whereas knockout of MDA5 had no marked effects (Fig. 5B). These results suggest that the RIG-I-VISA axis, rather than the MDA5-VISA axis, is required for mounting an effective innate immune response to EBIV in HEK293 cells. The essential role of RIG-I-VISA in driving the downstream response was further confirmed by rescue experiments, where re-expression of RIG-I in *RIG-I*^−/−^ HEK293 cells or VISA in *VISA*^−/−^ HEK293 cells restored IFN-β induction upon EBIV infection (Fig. S2A). Consistent with these findings, infection and replication of EBIV were enhanced in RIG-I- and VISA-deficient HEK293 cells, as evidenced by the elevated mRNA level of *S* segment and increased titers of progeny viruses in the culture supernatants (Fig. 5C and D). These findings suggest that RIG-I-VISA-mediated innate immune responses restrict EBIV replication in HEK293 cells. Similarly, knockout of RIG-I, but not MDA5, impaired the transcription of *IFNB1*, *CXCL10*, and *TNF* genes induced by EBIV in A549 cells (Fig. S2B). Furthermore, replication and production of EBIV were increased in RIG-I- but not MDA5-deficient A549 cells (Fig. S2C and D). These results demonstrate that RIG-I, signaling via VISA, is the essential cytoplasmic sensor responsible for triggering the type I IFN response to EBIV infection in human kidney and lung epithelial cell lines.

### Both RIG-I and MDA5 are involved in innate immune response to EBIV in Huh-7 and HCT116 cells

In contrast to our findings in HEK293 and A549 cells, results from Huh-7 cells revealed a different pattern of innate immune recognition of EBIV. We found that the knockout of both RIG-I and MDA5 only partially impaired the transcription of *IFNB1*, *CXCL10*, and *TNF* genes, as well as the phosphorylation of TBK1, IRF3, and p65 induced by EBIV in Huh-7 cells (Fig. 5E and F). However, expression of downstream genes and phosphorylation of these proteins induced by EBIV were completely blocked in VISA-deficient cells (Fig. 5E and F). Rescue experiments validated these findings, showing that re-expression of either RIG-I, MDA5, or VISA in their respective knockout cells restored IFN-β induction (Fig. S2E). In contrast, replication and production of EBIV were increased in both RIG-I- and MDA5-deficient Huh-7 cells, as indicated by elevated mRNA levels of *S* segment and higher titers of progeny viruses in the culture supernatants (Fig. 5G and H). Knockout of VISA resulted in an even greater increase in replication and production of EBIV compared to the knockout of RIG-I or MDA5 (Fig. 5G and H). Similar results were obtained in HCT116 cells. Knockout of RIG-I or MDA5 partially inhibited EBIV-induced transcription of *IFNB1*, *CXCL10*, and *TNF* genes, which was completely abolished in VISA-deficient HCT116 cells (Fig. S2F). Enhanced EBIV production was observed in these cells, where viral replication was higher in VISA-deficient cells than in RIG-I- and MDA5-deficient cells (Fig. S2G and H). These results suggest that RIG-I and MDA5 are functionally redundant upstream of VISA in the innate immune response to EBIV in human liver and colon carcinoma cell lines.

### Specificity of recognition of EBIV RNA species by RIG-I and MDA5

**Fig 6:**
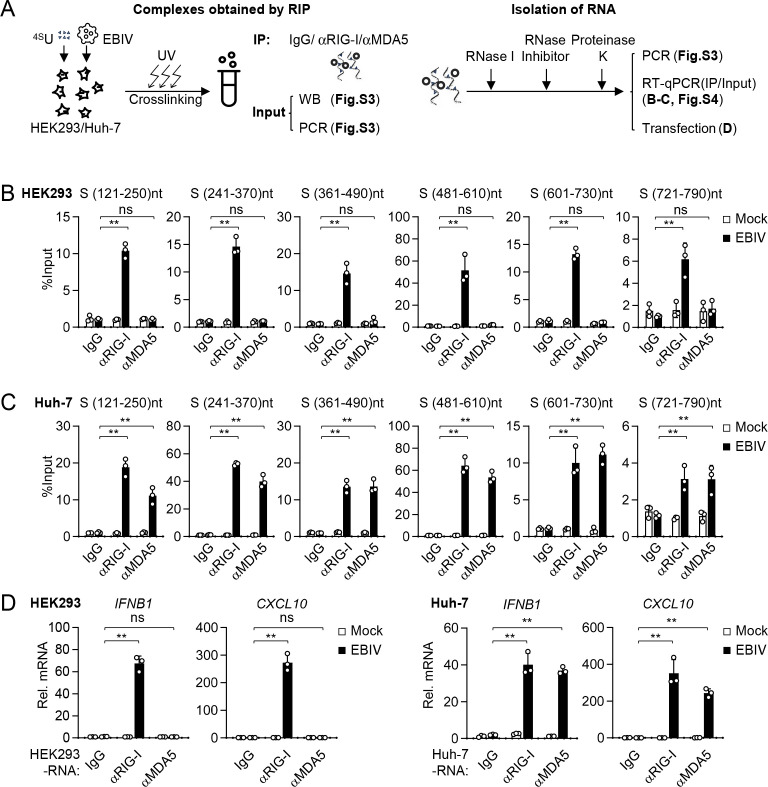
Recognition of EBIV RNA S segment by RIG-I and MDA5. (**A**) Schematic representation of the experimental procedure for PAR-CLIP analysis. (**B–D**) HEK293 (**B**) and Huh-7 (**C**) cells were pretreated with 4SU (100 µM) for 12 hours before infection with EBIV (MOI = 10) for 12 hours. Then, the cells were exposed to 0.15 J/cm^2^ 365 nm UV for 10 minutes. Cells were harvested, and endogenous RIG-I and MDA5 were immunoprecipitated with their respective antibodies. Bound RNA was extracted for RT-qPCR analysis with specific primers targeting the indicated segment (**B–C**). The “% input” represents the amount of a specific RNA amplicon in the IP fraction relative to its amount in the total input RNA sample (Fig. S3C and D) prior to immunoprecipitation. Results with other examined primers targeting EBIV RNA were shown in Fig. S4A and B. Bound RNA was transfected into HEK293 cells to test its immunostimulatory activity (**D**). nt, nucleotides. Data are shown as mean ± SD (*n* = 3 independent cell samples, **B–D**). Statistical significance was determined by one-way ANOVA. *P < 0.05; **P < 0.01. ns, not significant.

vRNA or its replication intermediates are well-characterized PAMP that triggers RLR signaling (32). We aimed to determine the specific EBIV RNA species recognized by RIG-I and MDA5. Similar to other bunyaviruses, EBIV possesses a negative-sense RNA genome consisting of three segments: a large (L) segment encoding RNA-dependent RNA polymerase (RdRP), a medium (M) segment encoding glycoproteins, and a small (S) segment encoding the nucleocapsid protein and non-structural proteins (4). We performed photoactivatable ribonucleoside–enhanced crosslinking and immunoprecipitation (PAR-CLIP) using antibodies against RIG-I or MDA5 in EBIV-infected Huh-7 and HEK293 cells, followed by PCR and RT-qPCR analysis (Fig. 6A). In HEK293 cells, where RIG-I is the primary PRR for EBIV, RIG-I bound specifically to the S segment, but not to the M or L segments (Fig. S3A). Further experiments indicated that RIG-I predominantly bound to the 121–790 region of the S segment in HEK293 cells (Fig. 6B; Fig. S3C and S4A). MDA5 did not bind significantly to any segment in HEK293 cells (Fig. 6B; Fig. S3A and S4A), which was not affected by type I IFN pretreatment (Fig. S4C). In Huh-7 cells, where both RIG-I and MDA5 contribute to sensing of EBIV, both receptors bound to the S segment but not M or L segments (Fig. S3B), mapping predominantly to the nt 121–790 region (Fig. 6C; Fig. S3D and S4B). Furthermore, RNA immunoprecipitated by RIG-I from HEK293 cells, or by either RIG-I or MDA5 from Huh-7 cells, induced transcription of *IFNB1* and *CXCL10* genes upon transfection into cells (Fig. 6D). These results indicate that RLRs preferentially recognize RNA derived from the EBIV S segment, thereby initiating antiviral innate immune responses.

**Fig 7:**
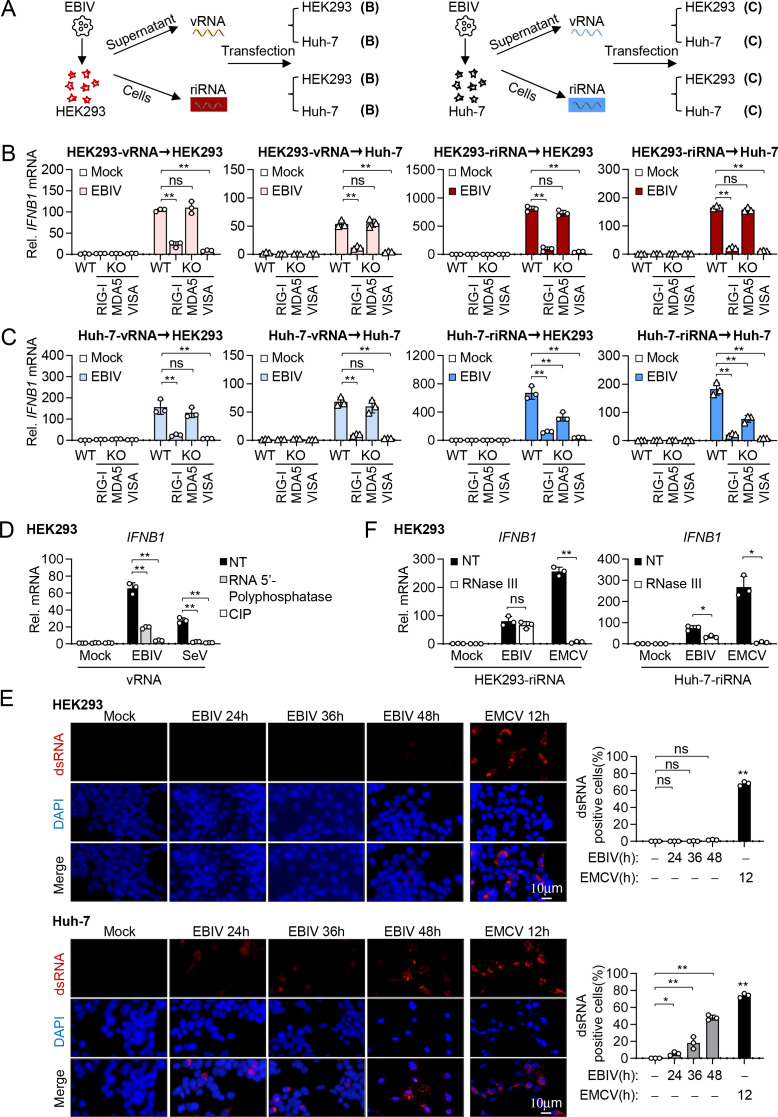
Distinct properties of EBIV RNA replication intermediates dictate the specificity of RLR utilization. (**A**) Schematic representation of the experimental procedure for B and C. (**B and C**) qPCR analysis of *IFNB1* in the indicated cells transfected with the indicated RNA (2 µg/mL) from HEK293 (B) or Huh-7 (C) cells for 12 hours. (**D**) qPCR analysis of *IFNB1* in HEK293 cells transfected with the indicated vRNA (0.5 mg/mL) for 12 hours after 5’-polyphosphatase (20U, 30 minutes at 37°C) and CIP (10U, 15 minutes at 37°C, 2 minutes at 80°C) treatment. (**E**) Immunostaining of dsRNA (red) in HEK293 and Huh-7 cells infected with EBIV (MOI = 1) or EMCV (MOI = 1) for the indicated times. Scale bar, 10 µm. The percentage of dsRNA-positive cells (J2 antibody) relative to total cells was quantified using ImageJ software. (**F**) qPCR analysis of *IFNB1* in HEK293 cells transfected with the indicated RNA (0.5 µg/mL) from HEK293 or Huh-7 cells for 12 hours after RNase III treatment (15U, 60 minutes at 37°C). Data are shown as mean ± SD (*n* = 3 independent cell samples, **B–F**). Statistical significance was determined by one-way ANOVA (**B–D**) or unpaired two-tailed Student’s *t*-test (**E–F**). *P < 0.05; **P < 0.01. ns, not significant.

We next determined the specificity of RIG-I and MDA5 in sensing EBIV genome RNA (vRNA) and replication intermediate RNA (riRNA). We extracted RNA from EBIV virions in culture medium (vRNA), or from infected cells, which mostly contain viral replication intermediates (riRNA). Transfecting these RNAs into HEK293 or Huh-7 WT or knockout cells revealed distinct patterns (Fig. 7A). Both vRNA and riRNA derived from EBIV-infected HEK293 cells strongly induced IFNB1 transcription upon transfection, an effect dependent on RIG-I and VISA, but not on MDA5 (Fig. 7B). Similarly, vRNA derived from infected Huh-7 cells activated responses solely via RIG-I and VISA (Fig. 7C). However, riRNA derived from infected Huh-7 cells induced *IFNB1* transcription, which was partially impaired by deficiency of either RIG-I or MDA5, and completely abolished by VISA deficiency in both recipient cell types (Fig. 7C). These results suggest that incoming EBIV vRNA primarily activates RIG-I, while replication in Huh-7 cells generates distinct RNA species capable of activating both RIG-I and MDA5.

It has been reported that the recognition of viral RNA by RIG-I requires a 5’ triphosphate or bisphosphate motif (15, 33, 34). Removing all phosphate groups using calf intestinal phosphatase (CIP) abolished transcription of the *IFNB1* gene induced by vRNA of EBIV or Sendai virus (SeV) (Fig. 7D), an ssRNA virus that requires 5’ triphosphate to activate RIG-I signaling (20) in HEK293 cells. Furthermore, treatment with 5’-polyphosphatase, an enzyme that sequentially removes the γ- and β-phosphates but does not affect the α-phosphate group, dramatically inhibited transcription of the *IFNB1* gene by EBIV vRNA (Fig. 7D). These results suggest that the 5' triphosphate or bisphosphate motif present in EBIV vRNA is required for RIG-I activation.

Previously, it has been shown that MDA5 preferentially senses long irregular dsRNA that presumably exists in a complex higher-order configuration (18, 19). Since Huh-7-derived riRNA activated MDA5 while HEK293-derived riRNA did not, we investigated whether these cells have differential formation of viral dsRNA intermediates following EBIV infection. Immunofluorescence analysis using a dsRNA-specific antibody revealed detectable dsRNA accumulation in EBIV-infected Huh-7 and HCT116 cells, increasing over time (24–48 hours), but dsRNA was barely detected in infected HEK293 or A549 cells at any time point (Fig. 7E; Fig. S5A). As positive controls, encephalomyocarditis virus (EMCV), a small non-enveloped single-stranded RNA virus that generates dsRNA during infection (20, 35), induced strong dsRNA staining in all cell types (Fig. 7E; Fig. S5A). Furthermore, dsRNA immunoprecipitation from infected Huh-7 cells followed by RT-qPCR confirmed the enrichment of viral S segment RNA in the dsRNA fraction (Fig. S5B). In contrast, no significant enrichment of viral S RNA was observed in infected HEK293 cells (Fig. S5B). Importantly, the host mitochondrial RNA MT-ND1 was not enriched by dsRNA-specific antibody in either cell line under infected conditions (Fig. S5B). These results indicate that the dsRNA accumulating in Huh-7 cells during EBIV infection is predominantly of viral origin rather than abundant host RNAs. Consistently, treatment of riRNA from EBIV-infected Huh-7 cells with dsRNA-specific RNase III inhibited its ability to induce *IFNB1* transcription (Fig. 7F). In contrast, treatment of riRNA from EBIV-infected HEK293 cells with RNase III had no marked effects on transcription of the *IFNB1* gene (Fig. 7F). In these experiments, treatment of riRNA from both EMCV-infected Huh-7 and HEK293 cells with RNase III abolished its ability to induce transcription of the *IFNB1* gene (Fig. 7F). Collectively, these results suggest that EBIV infection leads to the accumulation of immunostimulatory viral dsRNA intermediates specifically in Huh-7 and HCT116 cells, which are subsequently sensed by MDA5.

## DISCUSSION

EBIV is a recently identified virus within the Bunyavirales order (2), a family of viruses known for their segmented RNA genomes and their potential to cause severe diseases in humans and animals. Recent studies have demonstrated that EBIV can successfully infect several cell lines from different species, including mosquitoes, rodents, monkeys, and humans (4, 5). Serological surveys among local populations have indicated positive antibody responses against EBIV, highlighting a potential public health risk (4). However, the cellular and tissue tropisms of EBIV and the mechanisms by which it triggers innate immune responses remain poorly understood. Here, our findings reveal that EBIV infects and replicates in various human cell lines, including U-87 MG, Huh-7, HEK293, HeLa, SW-13, HCT116, HUVEC, and A549. Conversely, EBIV exhibits minimal infection in human monocytes, such as THP-1, U937, and PBMC, as well as murine macrophage BMDMs, but efficient infection in BMDCs. Furthermore, intraperitoneal injection of EBIV results in infection in several murine organs, including the kidney, spleen, intestine, brain, lung, liver, and lymph nodes. These results suggest that EBIV can infect a wide range of non-immune cell types in humans and mice but selectively infects certain immune cells, indicating the presence of specific receptors or entry pathways in a broad range of cell types.

Our findings demonstrate that multiple PRR signals are involved in antiviral innate immune responses to EBIV infection *in vitro* and *in vivo*. Our *in vitro* studies using knockout human cell lines delineated cell type-specific RLR sensing. In HEK293 and A549 cells, RIG-I signaling via VISA was essential for inducing type I IFN and inflammatory responses upon EBIV infection, while MDA5 appeared dispensable. This dominant role of RIG-I aligns with its established function in sensing negative-sense RNA viruses (36). In contrast, in Huh-7 and HCT116 cells, both RIG-I and MDA5 contributed to the induction of type I IFN and inflammatory responses. Deficiency of either sensor resulted in a partial reduction of the response, whereas VISA knockout completely abolished it, suggesting functional contribution and potential cooperation or redundancy between RIG-I and MDA5 upstream of VISA in these specific cell types. Our *in vivo* studies using knockout mice highlighted the physiological relevance and complexity of PRR engagement in controlling EBIV pathogenesis. Consistent with VISA being the central RLR adaptor, VISA deficiency resulted in the most severe phenotype, characterized by rapid mortality, broadly increased viral loads across multiple organs (spleen, liver, kidney, lung, and brain), widespread tissue pathology, and severely impaired systemic cytokine responses. RIG-I deficiency also led to high susceptibility, significantly increased viral loads in most organs tested, severe pathology (especially in the spleen and lung), and broadly impaired systemic cytokine production, confirming its major protective role i*n vivo*. MDA5 deficiency conferred increased susceptibility (though less severe than RIG-I or VISA knockout), higher viral loads/titers primarily restricted to the spleen and liver, and significantly reduced serum IFN-β, IP-10, and IL-6, indicating a more restricted, organ-specific role for MDA5 *in vivo*. Notably, the observation that VISA deficiency caused severe pathology in the liver and kidney, while individual RIG-I or MDA5 deficiency did not significantly increase pathology scores in these specific organs, supports the possibility of complementary or redundant functions between RIG-I and MDA5 in limiting immunopathology *in vivo*, particularly within the liver and kidney. Alternatively, this finding suggests that other immune receptors may be involved in sensing EBIV. Beyond the RLR pathway, our *in vivo* data revealed a critical, non-redundant role for TLR7 in host defense. *Tlr7*^−/−^ mice exhibited significantly increased susceptibility to lethal EBIV infection, associated with increased viral loads specifically in the spleen, lung, and liver and exacerbated pathology scores in the spleen and lung. These data suggest TLR7 contributes to viral control in specific tissues. Mechanistically, this pathway was distinct from RLRs; TLR7/MyD88 deficiency did not impact systemic IFN-β or IP-10 levels or their gene induction in BMDCs. Instead, TLR7 and MyD88 were essential for producing key inflammatory cytokines TNF-α and IL-6, both systemically and in BMDCs. This clearly distinguishes the role of TLR7-MyD88, likely operating primarily in DCs to drive inflammation, from the RLR-VISA pathway mediating the main antiviral IFN/ISG response.

The molecular basis for this differential RLR engagement appears linked to the specific viral PAMPs generated or recognized in different cellular contexts. Our PAR-CLIP experiments indicate that RLRs preferentially bind RNA derived from the EBIV S segment. Further investigation revealed that incoming vRNA primarily activates RIG-I, likely via its 5' triphosphate or bisphosphate motif, as demonstrated by transfection and phosphatase treatment experiments. However, riRNA derived from infected cells, particularly from Huh-7 cells, could activate both RIG-I and MDA5. This dual activation by Huh-7 riRNA correlated with the detectable accumulation of dsRNA intermediates in Huh-7 and HCT116 cells, but not in HEK293 or A549 cells, during EBIV infection. This accumulating dsRNA was confirmed to be predominantly of viral origin (S segment) rather than host mitochondrial RNA and was functionally relevant, as the immunostimulatory activity of Huh-7 riRNA was sensitive to RNase III digestion. These findings suggest that certain cellular environments, like those in Huh-7 and HCT116 cells, permit the formation or accumulation of sufficient viral dsRNA intermediates during EBIV replication to engage MDA5 signaling, complementing RIG-I sensing. Despite limitations in precisely separating vRNA from riRNA using cellular fractionation, the data collectively suggest that incoming vRNA is sensed by RIG-I and replication intermediates (including dsRNA in specific cells) are sensed by RIG-I and/or MDA5. The underlying reasons for cell type-specific dsRNA accumulation likely involve differences in intrinsic host factors regulating dsRNA stability or processing, such as RNA-binding proteins, helicases, or cellular ribonucleases, representing an important area for future investigation. Alternatively, the differential RLR engagement could be determined by the stoichiometry of viral PAMPs relative to endogenous host RNA-binding proteins, some of which may be IFN-inducible. In this model, MDA5 activation might only occur when dsRNA levels surpass the binding capacity of these competing cellular factors. Thus, cell type-specific differences in the expression of these competing proteins could be a key determinant of MDA5 engagement.

This study has certain limitations. While providing valuable mechanistic insights, immortalized cell lines do not fully recapitulate the complexity of primary cells or tissue environments. Our method for distinguishing vRNA and riRNA relied on cellular fractionation and has inherent limitations. Future studies employing more sophisticated techniques to precisely identify the structure and origin of RLR ligands, and to pinpoint the specific host factors responsible for differential dsRNA accumulation, will be essential to fully elucidate the mechanisms of EBIV sensing. Many bunyaviruses suppress host innate immune responses by their NSs proteins (37–40), and functionally characterizing the EBIV NSs protein will further deepen our understanding.

In conclusion, our studies have identified the cell tropisms of EBIV, a newly discovered orthobunyavirus. Our findings have elucidated the immune recognition mechanisms of EBIV by different PRRs in multiple cell types *in vitro* and in mice, which are determined by production of distinct PAMPs and intrinsic cellular factors during EBIV infection. Our study lays the foundation for understanding the mechanisms of EBIV-host interactions and provides a prototypic model on how RNA viruses are differentially sensed by distinct PRRs for complicated innate immune and inflammatory responses *in vivo*.

## MATERIALS AND METHODS

### Mice

*Rig-I*^flox/flox^ (S-CKO-07307) mice were purchased from Cyagen. *Mda5* ^−/−^(T013946), *Tlr7* ^−/−^(T006737), and *Tlr8*^−/−^ (T054108) mice were purchased from GemPharmatech. *cGas*^−/−^, *Mita*^−/−^, and *Visa*^−/−^mice were kindly provided by Prof. Hong-Bing Shu (Wuhan University) as previously described (30, 41). *Myd88*^−/−^ mice were kindly provided by Dr. Chunsheng Dong (Soochow University). Primer information for identification of different gene knockout mice is presented in the Table S1. All mice were housed in groups of five mice per cage under a 12-hour light/dark cycle in a temperature-controlled specific pathogen-free (SPF) room (23–25°C, relative humidity of 40%–70%) with free access to water and food. At the experimental endpoint, animals were euthanized by cervical dislocation after isoflurane anesthesia. Viral infection experiments were performed in the ABSL-2 facility at the Wuhan Institute of Virology.

### Cells and viruses

Huh-7, HUVEC, and A549 cell lines were obtained from the National Virus Resource Center (NVRC, Wuhan, China). U-87 MG, HEK293, SW-13, HeLa, HCT116, THP-1, and U937 cell lines were obtained from the American Type Culture Collection (ATCC; Manassas, VA, USA). Huh-7, HUVEC, A549, U-87 MG, HEK293, SW-13, HeLa, and HCT116 were cultured in Dulbecco’s modified Eagle’s medium (DMEM) (Gibco). THP-1 and U937 cells were cultured in RPMI 1640 medium (Hyclone). Bone marrow cells were isolated from the tibiae and femora. BMDCs were generated from mouse bone marrow cells induced by GM-CSF *in vitro*. In all cases, the medium was supplemented with 10% (vol/vol) fetal bovine serum (FBS) and 1% (vol/vol) penicillin–streptomycin (Gibco). The primary MEF and MLF cells were isolated and cultured as previously described (30, 42). Primary human PBMCs were isolated from whole blood of healthy donors with SepMate−15 (STEMCELL Technologies), according to the manufacturer’s instructions as previously described (43). Cells were maintained at 37°C in a humidified incubator containing 5% CO_2_. All cell lines were tested and found to be free of mycoplasma contamination using the MycoBlue Mycoplasma Detector Kit (#D101, Vazyme).

EBIV (Cu-XJ20 isolate) and rEBIV/eGFP/S were provided by Dr. Han Xia (Wuhan Institute of Virology). EMCV and SeV have been previously described (37).

### Reagents and antibodies

Lipofectamine 2000 (Thermo Scientific, 11668), polybrene transfection reagent (Specialty Media, TR-1003-G), protein G sepharose (Cytiva, 17061805), 4-thiouridine (MCE, HY-W011793), favipiravir (TargetMol, T6833), RNase I (Thermo Scientific, EN0601), RNase inhibitor (Invitrogen, AM2694), protein K (Thermo Scientific, 25530-049), RNA 5’ polyphosphatase (Biosearch, RP8092H), Quick CIP (NEB, M0525S), and RNaseIII (Invitrogen, AM2290) were purchased from the indicated companies. Information on the commercially available antibodies used in this study is provided in the Table S2.

### CRISPR/Cas9 knockout

Gene editing was performed using the CRISPR/Cas9 system. Briefly, double-stranded oligonucleotides corresponding to the target sequences were cloned into the lenti-CRISPR-V2 vector, which was co-transfected with the packaging plasmids psPAX2 and pMD2.G into HEK293 cells. Two days after transfection, lentiviruses were harvested and used to infect target cells in the presence of polybrene (8 µg/mL). The infected cells were selected with puromycin for at least seven days. The sequences of gRNAs are provided in Table S3. Mutation and deficiency of the target gene were confirmed by Sanger sequencing and immunoblots, respectively.

### RT-qPCR

Total RNA from the cells was isolated using RNAiso Plus (#9109, TaKaRa), and reverse transcription of 1 µg of RNA was conducted using a cDNA synthesis kit (#R222, Vazyme) according to the manufacturer’s instructions. For the extraction of viral RNA in cell culture supernatant, TaKaRa MiniBEST Viral RNA/DNA Extraction Kit Ver.5.0 was used (#9766, TaKaRa). RT-qPCR was performed as previously described. The threshold cycle (Ct) for the indicated genes was normalized to that of the housekeeping gene GAPDH and is presented as the relative mRNA level. Gene-specific primers used in this study are listed in Table S4.

### Immunoblots

Cells were lysed in lysis buffer (20 mM Tris-HCl, pH 7.4, 150 mM NaCl, 1 mM EDTA, 1% NP-40) supplemented with a complete protease inhibitor mixture (Targetmol) and incubated on ice for 15 minutes. Insoluble materials were removed by centrifugation. The lysates were fractionated by SDS-PAGE and transferred to a nitrocellulose filter membrane (Millipore).

### Analysis of dsRNA by confocal microscopy

HEK293 and Huh-7 cells were infected with EBIV and EMCV for the indicated time points. After infection, the cells were fixed and stained as previously described (44). The cells were incubated with the anti-dsRNA antibody (1:200) (Nordic Mubio, 10010200) for 1 hour. After washing, Alexa Fluor 555-conjugated secondary antibody was applied at a 1:2000 dilution for 1 hour. The nuclei were stained with DAPI for 2 minutes. Images were acquired using MSHOT microscope with a 40 × lens objective.

### Flow cytometry analysis for EBIV-GFP infected cells

The cells were infected with EBIV-GFP for the indicated times, harvested, and washed with PBS. The cells were then counted (10,000 cells per sample) and analyzed using flow cytometry. Data were analyzed and visualized using FlowJo software.

### TCID50 assay

Cells were plated in 96-well plates overnight before being subjected to TCID50 assays. Briefly, EBIV stock was serially diluted from 1:10 to 1:10^6^ with DMEM. Cells were incubated with 100 mL of each diluted virus stock for 1 hour. The virus was then removed, and 150 µL of DMEM containing 2% FBS was added to cells to maintain cell growth for 5 days. Cytopathic effects were observed, and viral titers were calculated using the Reed–Muench method.

### PAR-CLIP

Cells were infected with EBIV (MOI = 10) in the presence of 4SU (100 µM). The cells were washed with PBS and exposed to 0.15 J/cm^2^ 365 nm UV light to crosslink the labeled RNA to RNA-binding proteins. Cells were harvested and lysed in Nonidet P-40 lysis buffer (50 mM HEPES, 150 mM KCl, 1 mM NaF, 10 mM ZnCl_2_, 0.5% NP-40, 0.5 mM DTT, protease inhibitor, pH 7.5) for 10 minutes on ice. The lysate was cleared by centrifugation, and endogenous proteins were immunoprecipitated for 4 hours with the respective antibodies (1 µg/ mL). The immunoprecipitates were further digested with 100 U ml^−1^ RNase I. Beads were washed five times with high-salt wash buffer (50 mM HEPES, 500 mM KCl, 0.05% NP-40, 0.5 mM DTT, protease inhibitor, pH 7.5) and incubated with proteinase K (Thermo Scientific) for 30 minutes at 55°C. The RNA was isolated by phenol/chloroform/isoamyl alcohol extraction and subjected to further RT-qPCR analysis.

### Viral infection in mice

For measurement of the survival rate, age- and sex-matched mice were infected i.p. with EBIV (1 × 10^3^ PFU per mouse) and monitored daily for up to 10 days. For *in vivo* viral infection, mice were i.p. infected with EBIV (1 × 10^3^ PFU per mouse). Lungs and livers from the control or virus-infected mice were harvested 48 hours post-infection for histological analysis. The tissue mRNA levels of the indicated genes were determined by RT-qPCR assays. Mouse serum was collected 48 hours post-infection to measure cytokine production by ELISA.

### Histological and immunohistochemical analysis

Tissue samples were fixed in 4% paraformaldehyde, dehydrated in graded ethanol, cleared in xylene, and embedded in paraffin. Serial sections (2 µm) were subjected to Hematoxylin and Eosin (H&E) staining following standard procedures. Histopathological parameters, including tissue damage/necrosis, inflammatory cell infiltration, vacuolization, and hemorrhage, were assessed and semi-quantitatively scored on a scale of 0 to 3, with the following criteria: 0 denotes no abnormality; 1 denotes mild change (necrosis  <10% of the tissue area, inflammatory cells  <  10% of the field of view, vacuolization <25% of the tissue area, or petechial hemorrhage); 2 denotes moderate change (necrosis or infiltration 10–30%, vacuolization 25–50%, or patchy hemorrhage); and 3 denotes severe change (necrosis or infiltration  > 30%, vacuolization > 50%, or massive hemorrhage).

For immunohistochemistry (IHC), sections were repaired antigenically by high pressure treatment in repair solution (pH 9.0 EDTA). Sections were then incubated with a primary mouse anti-EBIV NP antibody (1:2,000) overnight at 4°C, followed by incubation with a goat anti-mouse IgG-HRP secondary antibody (Abcam, ab205719, 1:2000) at 37°C for 45 minutes. Immunoreactivity was visualized using DAB chromogenic reagent (DAB-4033, diluted 1:20), and slides were counterstained with hematoxylin. Digital images of stained sections were captured using a NanoZoomer S360 scanner.

### Enzyme-linked immunosorbent assay (ELISA)

Concentrations of cytokines in mouse serum were measured using mouse IFN-β (Biolegend, 439407), IP-10 (4A Biotech, CME0016), TNF-α (Biolegend, 430904), and IL-6 (Biolegend, 431304) ELISA Kit according to the manufacturer’s protocol.

### Statistics and reproducibility

For *in vivo* mouse experiments, data represent biological replicates (*n* ≥ 4), where *n* denotes samples from distinct individual mice, and statistical analyses were conducted on these biological replicates. For *in vitro* cell culture experiments, data shown are from a representative experiment performed with multiple independent cell samples (*n* ≥ 3 wells or dishes prepared and treated independently in parallel), which served as replicates for statistical analysis within that specific experiment. Differences between experimental and control groups were determined by unpaired two-tailed Student’s *t-*test (when two groups of data were compared) or one-way ANOVA analysis (when more than two groups of data were compared). Statistical analysis of survival curves was performed using the log-rank test (Mantel-Cox). The key findings were confirmed through independent biological repetitions (typically 2–3 times) with consistent results. Statistically analyzed data are expressed as mean ± standard deviation (S.D.). A *P* value < 0.05 was considered statistically significant. Statistical analysis was performed using Prism software (GraphPad version 9.5.1).

## Data Availability

All data supporting the findings of this study are available within the article and its supplemental material or can be obtained from the corresponding author upon reasonable request.

## SUPPLEMENTAL MATERIAL

Supplemental figures and tables jvi.00750-25-s0001.pdf Fig. S1 to S6, and Tables S1 to S5.

ASM does not own the copyrights to Supplemental Material that may be linked to, or accessed through, an article. The authors have granted ASM a non-exclusive, world-wide license to publish the Supplemental Material files. Please contact the corresponding author directly for reuse.
