# Supplementary material for: Sensing of Ebinur Lake virus by distinct pattern recognition receptors dictates cell-type specific innate immunity and pathogenesis
Source: J Virol. 2025 Sep 15;99(10):e00750-25. doi: 10.1128/jvi.00750-25 (PMC12548452; doi:10.1128/jvi.00750-25)
Supplement: Supplemental figures and tables — Fig. S1 to S6, and Tables S1 to S5. [file jvi.00750-25-s0001.pdf]

Supplementary Information

Sensing of Ebinur Lake Virus by distinct pattern recognition receptors dictates cell-type specific innate immunity and pathogenesis

Jia-Peng Zou[1,2], Su-Yun Wang[1], Han Xia[1,2], Zhi-Sheng Xu[1,2], Wei-Wei Luo[1,2,*], Yan-Yi Wang[1,2,*]

This PDF file includes:

Supplementary Figures 1 to 6

Supplementary Tables 1 to 5

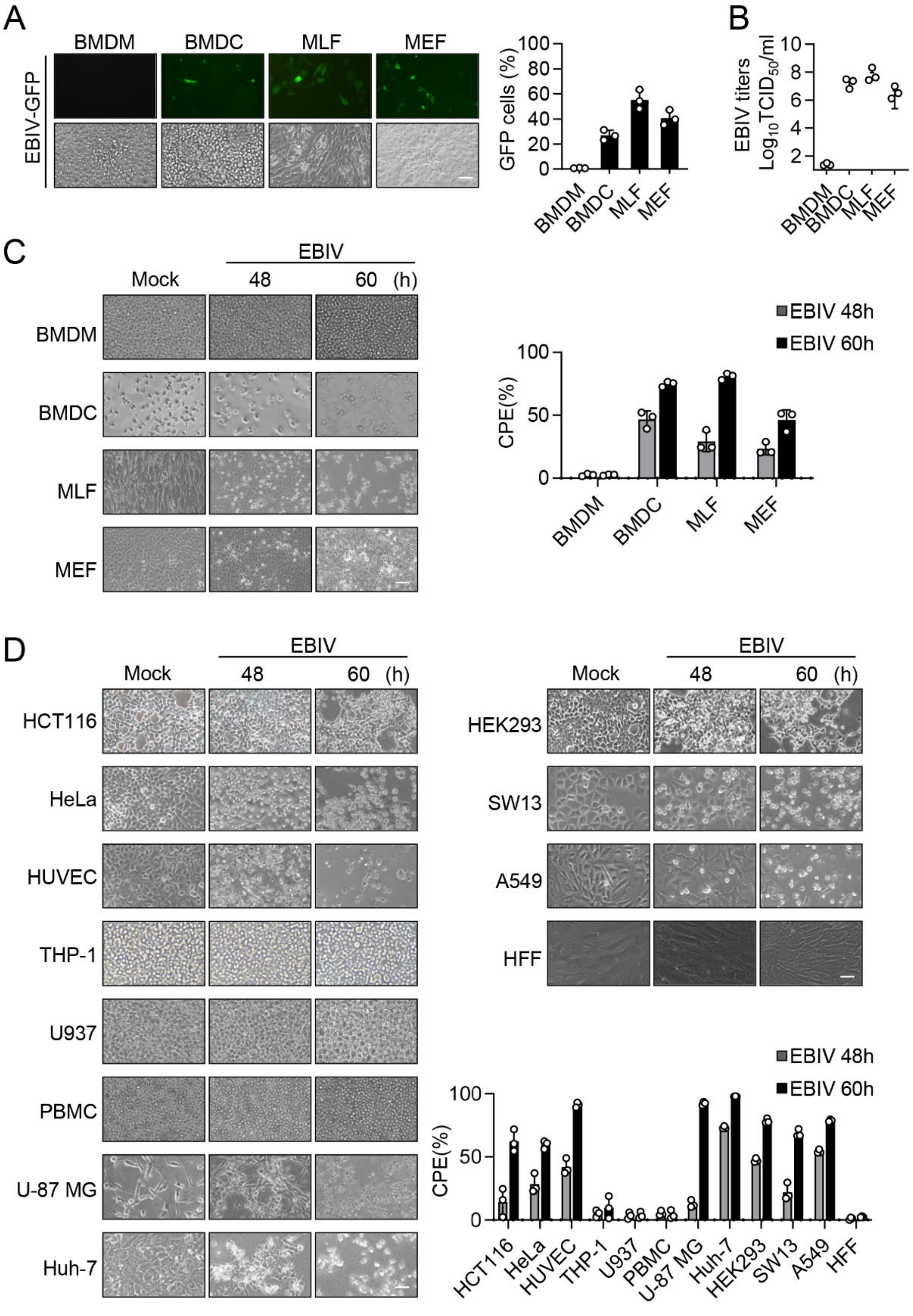
A
BMDM
BMDC
MLF
MEF
EBIV-GFP
GFP cells (%)
B
EBIV titers
Log10TCID50/ml
C
Mock
EBIV
48
60
(h)
EBIV 48h
EBIV 60h
CPE(%)
D
HCT116
HeLa
HUVEC
THP-1
U937
PBMC
U-87 MG
Huh-7
HEK293
SW13
A549
HFF

Supplementary Figure 1. EBIV exhibits broad cell tropism.

(A) Fluorescence microscopy (left) and flow cytometry quantification (right) of EBIV-GFP infection (MOI=1, 48 hours) in the indicated primary murine cells. Scale bar, 25μm.

(B) Progeny virus titers (TCID50) in supernatants from primary murine cells infected with EBIV (MOI=1) for 48 hours.

(C-D) Cytopathic effect (CPE) in EBIV-infected primary murine cells (C) and human cell lines (D) at 48 or 60 hours post-infection (MOI=1). Scale bar, 25μm. The graph shows quantification of CPE (%) by ImageJ.

Data are shown as mean ± SD (n=3 independent cell samples).

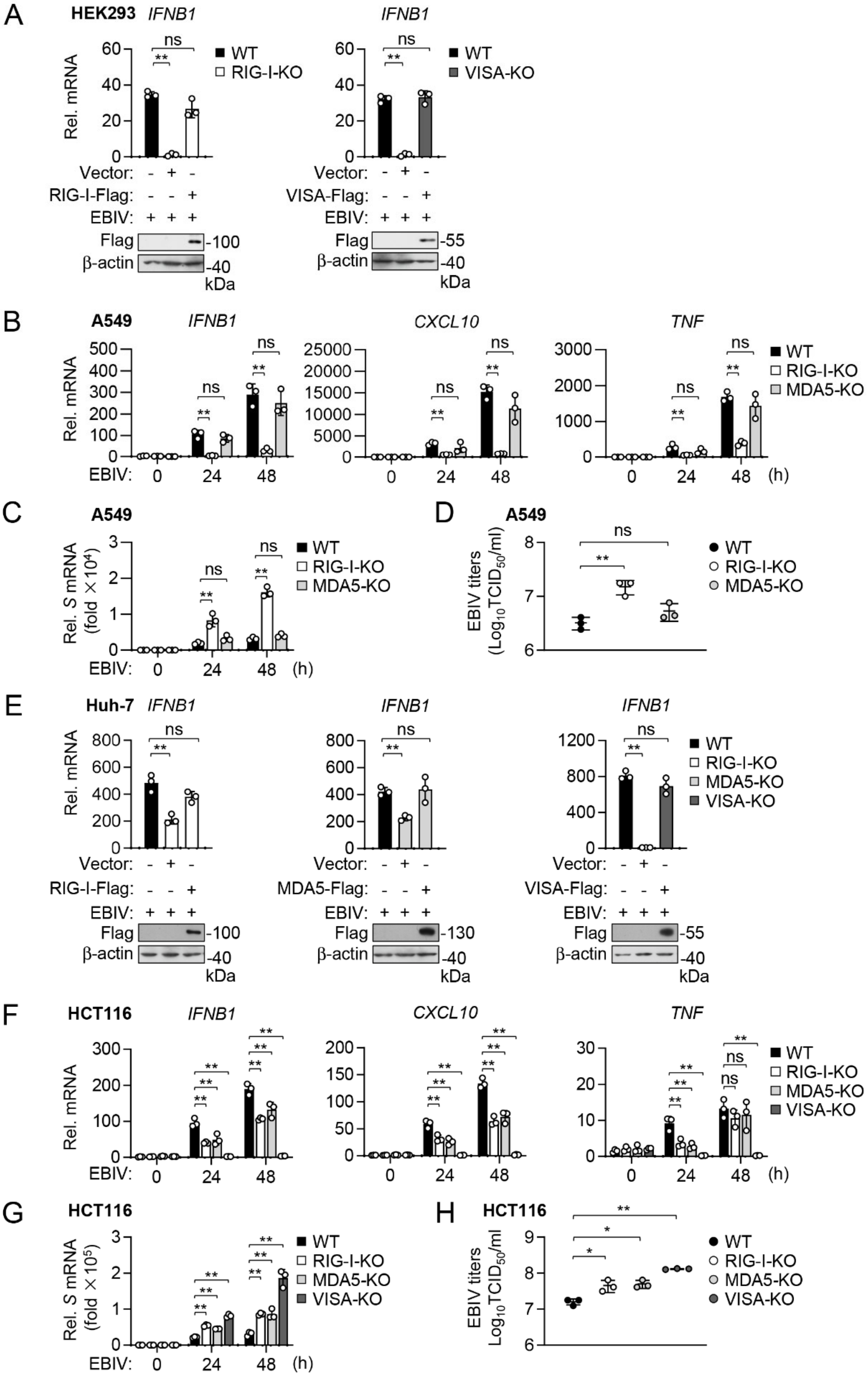
A
HEK293
IFNB1
Rel. mRNA
WT
RIG-I-KO
VISA-KO
Vector:
RIG-I-Flag:
VISA-Flag:
EBIV:
Flag
β-actin
-100
-55
-40
kDa
B
A549
IFNB1
CXCL10
TNF
MDA5-KO
(h)
C
Rel. S mRNA
(fold × 10^4)
D
EBIV titers
(Log10TCID50/ml)
E
Huh-7
MDA5-Flag:
-130
F
HCT116
G
(fold × 10^5)
H
ns
**
*

Supplementary Figure 2. Effects of knockout of RIG-I, MDA5 or VISA on innate immune response and EBIV replication.

(A) Rescue of IFN-β induction by EBIV (MOI=1, 24h) in RIG-I or VISA knockout HEK293 cells transfected with corresponding expression plasmids or empty vector (2μg/well, 24 hours). Upper: RT-qPCR for *IFNB1* mRNA. Lower: Immunoblot for expressed proteins and β-actin.

(B-D) RT-qPCR analysis of the indicated genes in A549 cells uninfected and infected with EBIV (MOI=1) for the indicated times (B, C). Production of EBIV progeny viruses in supernatants was measured by $TCID_{50}$ assay (D).

(E) Rescue of IFN-β induction by EBIV (MOI=1, 24h) in RIG-I, MDA5, or VISA knockout Huh-7 cells transfected with corresponding expression plasmids or empty vector (2μg/well, 24 hours). Upper: RT-qPCR for *IFNB1* mRNA. Lower: Immunoblot for expressed proteins and β-actin.

(F-H) RT-qPCR analysis of the indicated genes in HCT116 cells uninfected and infected with EBIV (MOI=1) for the indicated times (F, G). Production of EBIV progeny viruses in supernatants was measured by $TCID_{50}$ assay (H).

Data are shown as mean ± SD (n=3 independent cell samples, A-H), statistical significance was determined by one-way ANOVA. *, $p < 0.05$; **, $p < 0.01$. ns, not significant.

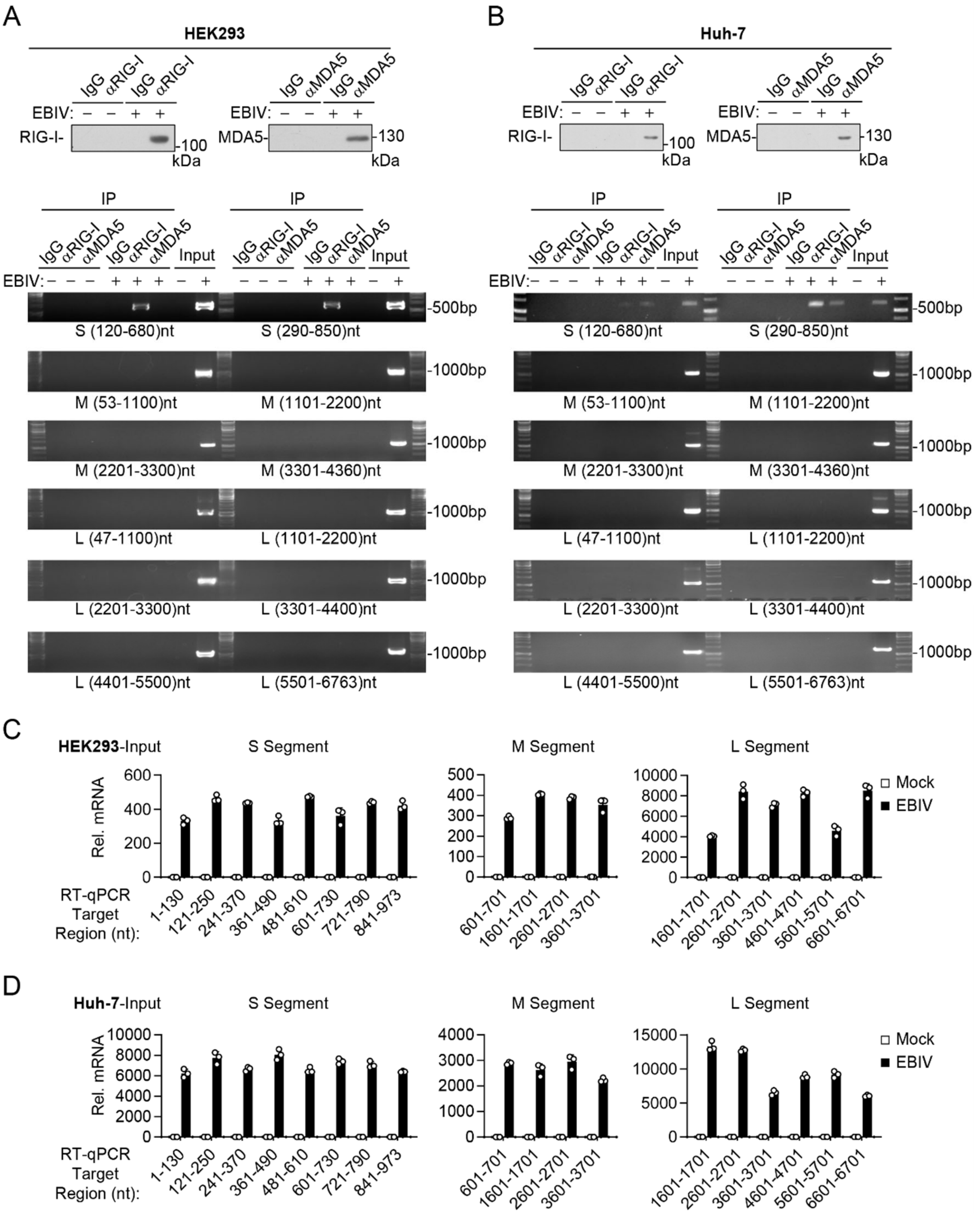
A
HEK293
IgG
αRIG-I
IgG
αRIG-I
EBIV: − − + +
RIG-I-
-100 kDa
IgG
αMDA5
IgG
αMDA5
EBIV: − − + +
MDA5-
-130 kDa
IP
IgG
αRIG-I
αMDA5
Input
EBIV: − − − + + + − +
-500bp
S (120-680)nt
S (290-850)nt
-1000bp
M (53-1100)nt
M (1101-2200)nt
M (2201-3300)nt
M (3301-4360)nt
L (47-1100)nt
L (1101-2200)nt
L (2201-3300)nt
L (3301-4400)nt
L (4401-5500)nt
L (5501-6763)nt
B
Huh-7
IgG
αRIG-I
IgG
αRIG-I
EBIV: − − + +
RIG-I-
-100 kDa
IgG
αMDA5
IgG
αMDA5
EBIV: − − + +
MDA5-
-130 kDa
IP
IgG
αRIG-I
αMDA5
Input
EBIV: − − − + + + − +
-500bp
S (120-680)nt
S (290-850)nt
-1000bp
M (53-1100)nt
M (1101-2200)nt
M (2201-3300)nt
M (3301-4360)nt
L (47-1100)nt
L (1101-2200)nt
L (2201-3300)nt
L (3301-4400)nt
L (4401-5500)nt
L (5501-6763)nt
C
HEK293-Input
S Segment
M Segment
L Segment
Rel. mRNA
Mock
EBIV
RT-qPCR Target Region (nt):
1-130
121-250
241-370
361-490
481-610
601-730
721-790
841-973
601-701
1601-1701
2601-2701
3601-3701
1601-1701
2601-2701
3601-3701
4601-4701
5601-5701
6601-6701
D
Huh-7-Input
S Segment
M Segment
L Segment
Rel. mRNA
Mock
EBIV
RT-qPCR Target Region (nt):
1-130
121-250
241-370
361-490
481-610
601-730
721-790
841-973
601-701
1601-1701
2601-2701
3601-3701
1601-1701
2601-2701
3601-3701
4601-4701
5601-5701
6601-6701

Supplementary Figure 3. PCR detection of EBIV RNA segments associated with RIG-I and MDA5.

(A-B) HEK293 (A) and Huh-7 (B) cells were pretreated with 100 μM 4SU for 12 hours before infection with EBIV (MOI=10) for 12 hours. Then the cells were exposed to 0.15 J/cm^2 365 nm UV for 10 min. Cells were harvested, and endogenous RIG-I and MDA5 were immunoprecipitated with the respective antibodies. Upper: Immunoblot confirming immunoprecipitation of endogenous RIG-I and MDA5. Lower: PCR analysis of immunoprecipitated RNA using primers targeting indicated regions of EBIV S, M, and L segments. nt, nucleotides.

(C-D) RT-qPCR analysis of input RNA from HEK293 (C) and Huh-7 (D) cells with primers targeting the indicated segments. nt, nucleotides.

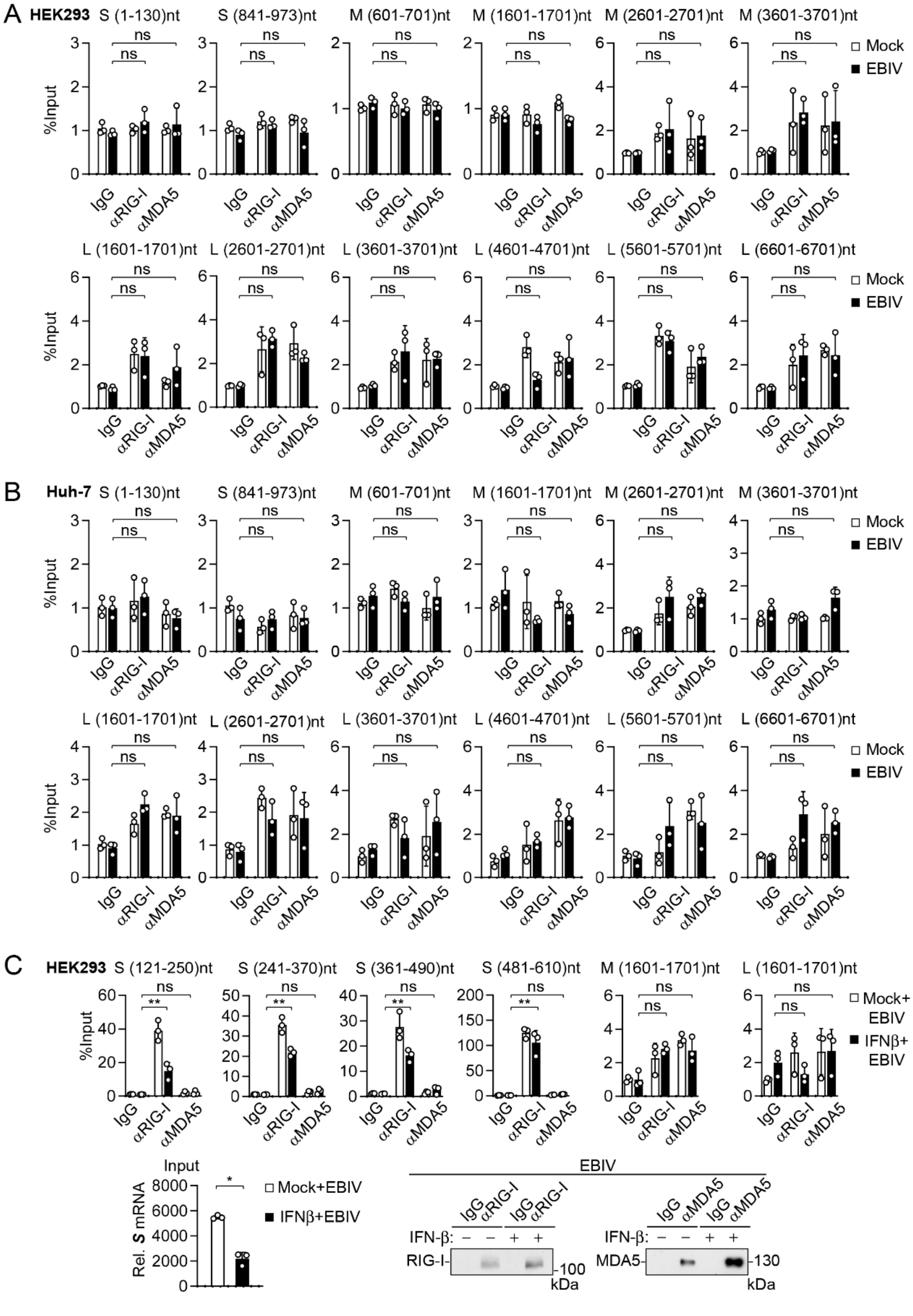
A
HEK293
S (1-130)nt
S (841-973)nt
M (601-701)nt
M (1601-1701)nt
M (2601-2701)nt
M (3601-3701)nt
L (1601-1701)nt
L (2601-2701)nt
L (3601-3701)nt
L (4601-4701)nt
L (5601-5701)nt
L (6601-6701)nt
%Input
ns
IgG
αRIG-I
αMDA5
□ Mock
■ EBIV
B
Huh-7
C
HEK293
S (121-250)nt
S (241-370)nt
S (361-490)nt
S (481-610)nt
**
□ Mock+ EBIV
■ IFNβ+ EBIV
Input
Rel. S mRNA
*
□ Mock+EBIV
■ IFNβ+EBIV
EBIV
IFN-β:
RIG-I
MDA5
100 kDa
130 kDa

Supplementary Figure 4. RT-qPCR detection of EBIV RNA segments associated with RIG-I and MDA5.

(A-B) HEK293 (A) and Huh-7 (B) cells were pretreated with 100 μM 4SU for 12 hours before infection with EBIV (MOI=10) for 12 hours. Then the cells were exposed to 0.15 J/cm^2 365 nm UV for 10 min. Cells were harvested, and endogenous RIG-I and MDA5 were immunoprecipitated with the respective antibodies. Pulldown of endogenous RIG-I and MDA5 was measured by RT-qPCR with specific primers targeting the indicated segment. The '% input' represents the amount of a specific RNA amplicon in the IP fraction relative to its amount in the total input RNA sample prior to immunoprecipitation. nt, nucleotides.

(C) PAR-CLIP analysis in HEK293 cells pretreated with Type I IFN (20 μM) and 4SU before EBIV infection (MOI=10, 12h). Upper: RT-qPCR analysis of bound RNA targeting indicated segments. Results shown as % input. Lower: RT-qPCR analysis of viral *S* gene in input. Immunoblot confirming RIG-I/MDA5 IP.

Data are shown as mean ± SD (n=3 independent cell samples, A-C), statistical significance was determined by one-way ANOVA. *, $p < 0.05$. ns, not significant.

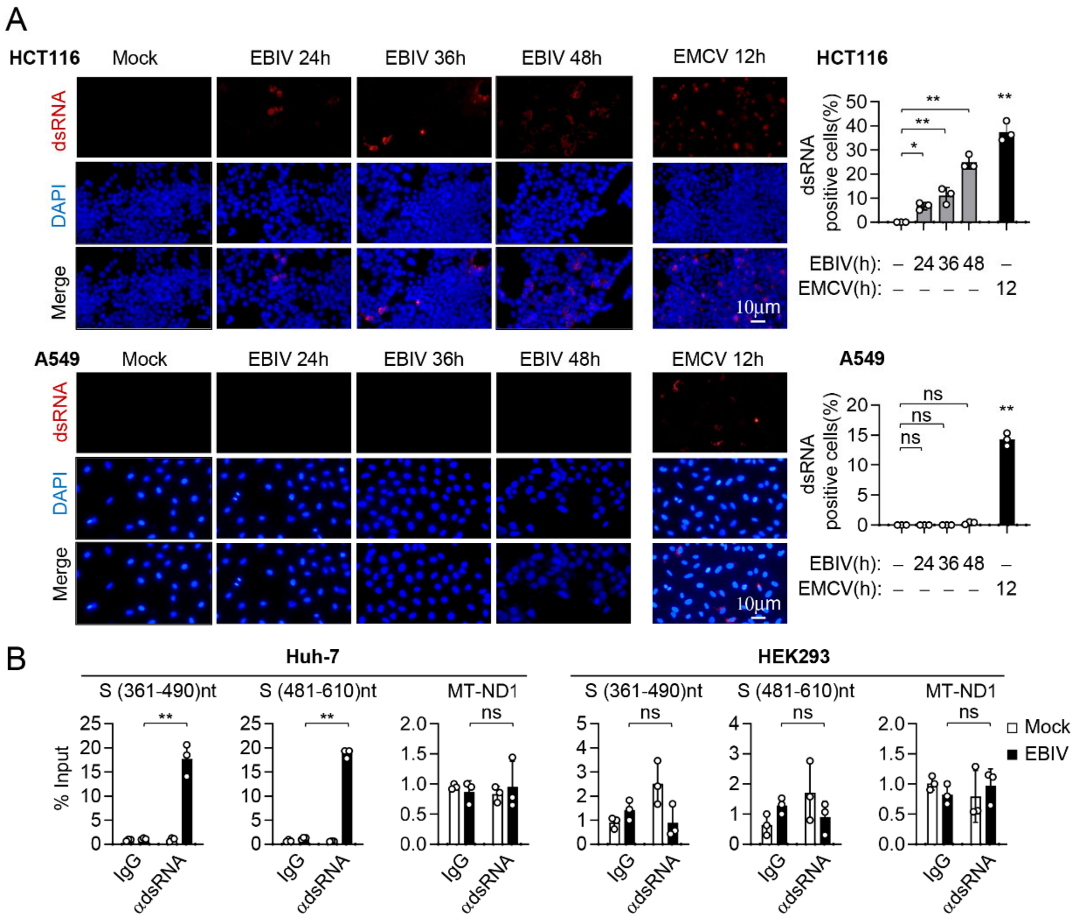

Supplementary Figure 5. EBIV infection induces the production of viral double-stranded RNA in HCT116 cells

(A) Immunofluorescence staining for dsRNA (red, J2 antibody) and nuclei (blue, DAPI) in HCT116 and A549 cells infected with EBIV (MOI=1) for the indicated times or EMCV (MOI=1, 12 hours). Scale bar, 10µm. Graphs show quantification of dsRNA-positive cells (%).

(B) RT-qPCR analysis of EBIV S segment RNA and host MT-ND1 RNA immunoprecipitated using an anti-dsRNA antibody (or IgG control) from Huh-7 and HEK293 cells infected with EBIV (MOI=1, 48 hours). Results shown as % input. nt, nucleotides.

Data are shown as mean ± SD (n=3, independent cell samples), statistical significance was determined by one-way ANOVA (A) or unpaired two-tailed Student's t-test (B). *, $p < 0.05$; **, $p < 0.01$. ns, not significant.

Supplementary Figure 6. Uncropped immunoblots

Fig.1F

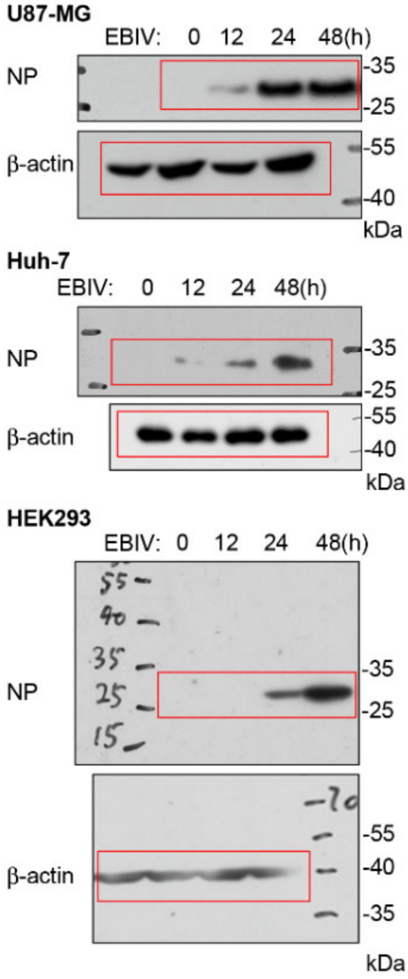

Fig.2A

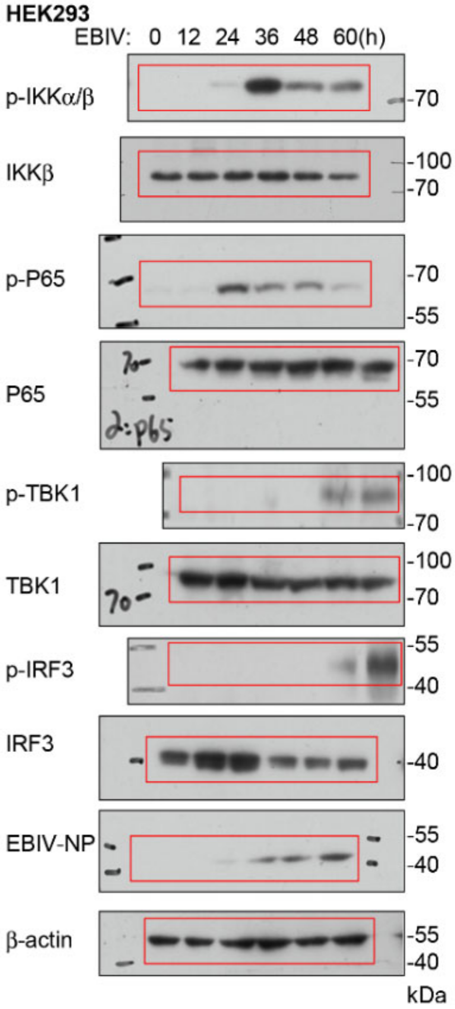

Fig.5B

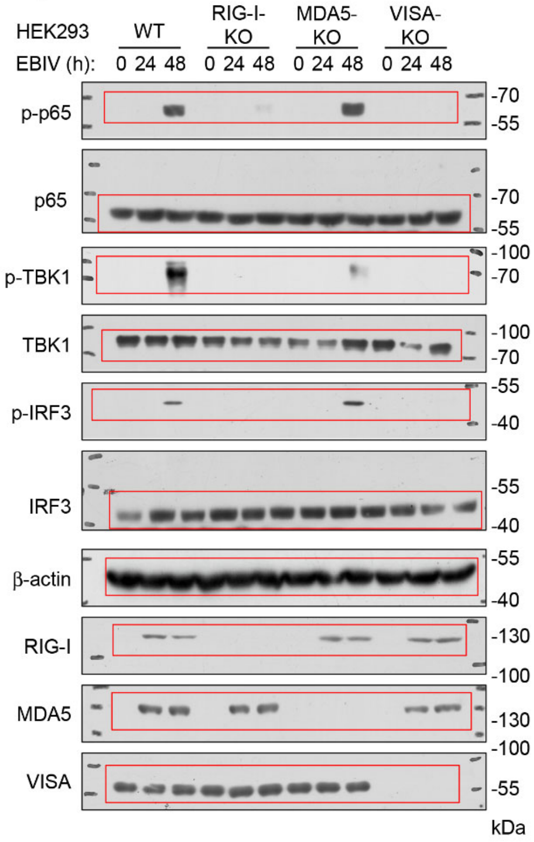
HEK293
WT
RIG-I-KO
MDA5-KO
VISA-KO
EBIV (h): 0 24 48 0 24 48 0 24 48 0 24 48
p-p65
p65
p-TBK1
TBK1
p-IRF3
IRF3
β-actin
RIG-I
MDA5
VISA
-70
-55
-100
-40
-130
kDa

Fig.5F

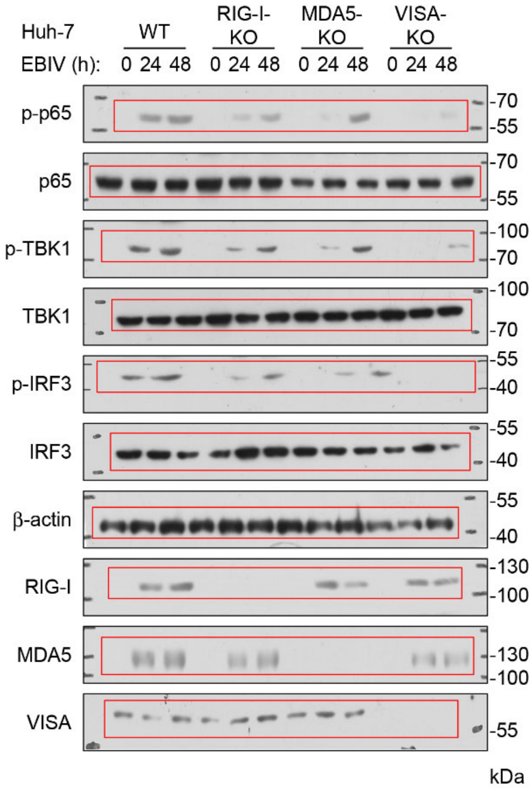
Huh-7
WT
RIG-I-KO
MDA5-KO
VISA-KO
EBIV (h): 0 24 48 0 24 48 0 24 48 0 24 48
p-p65
p65
p-TBK1
TBK1
p-IRF3
IRF3
β-actin
RIG-I
MDA5
VISA
-70
-55
-100
-40
-130
kDa

Fig.S2A

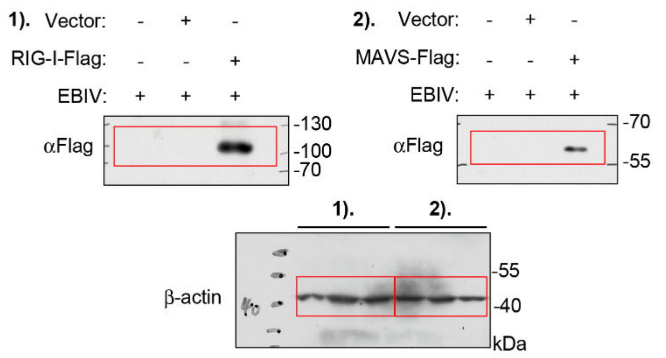

Fig.S2E

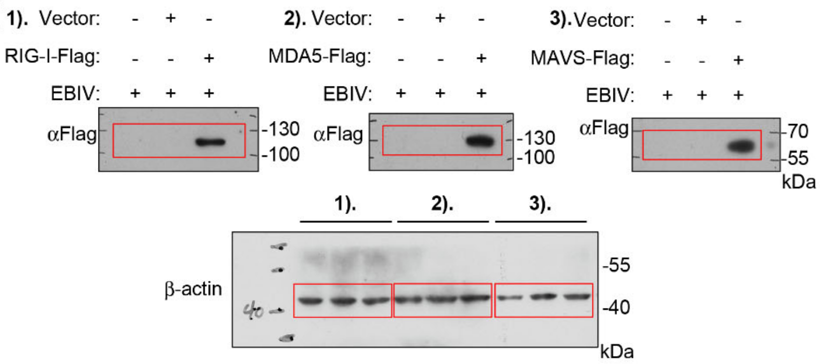

Fig.S3A

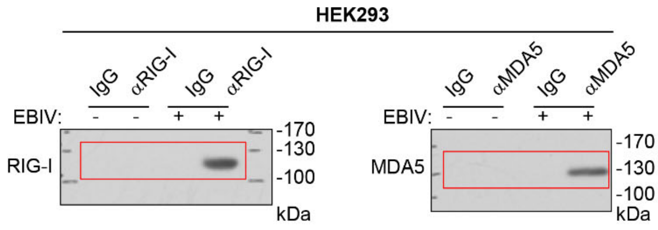

Fig.S3B

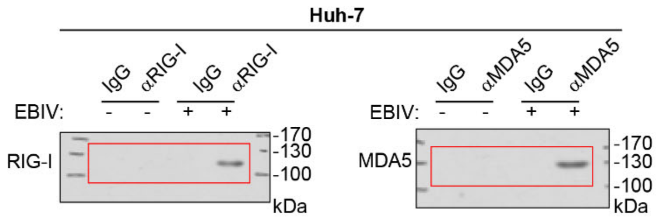

Fig.S4C

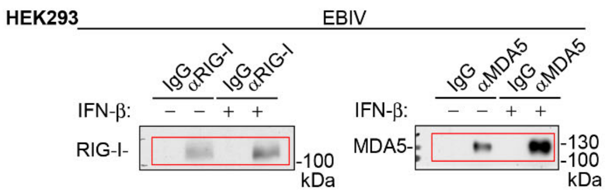

Supplementary Table 1. A list of primers for mouse genotyping

Primer Name	Sequence	Note
RIG-I(flox/flox)-	5'-AGGCCCAAGGATAATTGAAACCC -3'	PCR(F+R): Wild 289 bp, Mutant 417 bp
RIG-I(flox/flox)-	5'-CTGCCTGGCATCACATCACTAGTT -3'	
LysM-Cre-1	5'-CCCAGAAATGCCAGATTACG -3'	PCR (1+2+3): Wild 350bp, Mutant 700 bp
LysM-Cre-2	5'-CTTGGGCTGCCAGAATTTCTC -3'	
LysM-Cre-3	5'-TTACAGTCGGCCAGGCTGAC -3'	
MDA5-WT-F	5'-TAGAAGCCAGGGTCAAGAG -3'	PCR WT(F+R): Wild 328bp PCR KO(F+R): Mutant 247bp
MDA5-WT-R	5'-CTGGGGTATGCAGAGAGAATA -3'	
MDA5-KO-F	5'-CCAGAGTGGTAACAGTCAAGGAAGTG -3'	
MDA5-KO-R	5'-GGAGGCAGGAAAGGGTAGAGCAT -3'	
VISA-P1	5'-TAGCTGTGAGGCAGGACAGGTAAGG -3'	PCR(P1+P2+P3): Wild 250bp, Mutant 350bp
VISA-P2	5'-AGCCAAGATTCTAGAAGCTGAGAA -3'	
VISA-P3	5'-GTGGAATGTGTGCGAGGCCAGAGGC -3'	
cGAS-F	5'-ATATTTCCCCCTGTGTTGGA -3'	PCR(F+R1+R2): Wild 188bp, Mutant 298bp
cGAS-R1	5'-GTGCCAGGTGACACAACATC -3'	
cGAS-R2	5'-CGGATGGATGAACAAACAGA -3'	
MITA-F	5'-CTCCTAGACAGGTGCTGTAGGATG -3'	PCR (F+WT-R): Wild 420bp PCR(F+KO-R):
MITA-WT-R	5'-TGGAGACCACAGAGGGTTACCTG -3'	
MITA-KO-R	5'-AAGGGTTATTGAATATGATCGGA -3'	
Myd88-WT-F	5'-AGACAGGCTGAGTGCAAACTTGTGCTG -3'	PCR (WT-F+R): Wild 1200bp PCR (KO-F+R):
Myd88-KO-F	5'-ATCGCCTTCTATCGCCTTCTTGACGAG -3'	
Myd88-R	5'-AGCCTCTACACCCTTCTCTTCTCCACA -3'	
TRIF-WT-F	5'-CAAGATTGGACTTCACCTGGGTCCTTA -3'	PCR (WT-F+R): Wild 1300bp PCR (KO-F+R):
TRIF-KO-F	5'-CTAAAGCGCATGCTCCAGACTGCCTTG -3'	
TRIF-R	5'-CTGACACACTGTGTACTTACTAGGTGC -3'	
TLR7-F1	5'-TGCAACAGCATGTGAAACATGAC -3'	PCR (F1+R1): Mutant 306bp PCR (F2+R2): Wild 444bp
TLR7-R1	5'-GTAGGGAACTTTCAGGATAGCATTTG -3'	
TLR7-F2	5'-TAATATGAGGCTGGCTCACCCAG -3'	
TLR7-R2	5'-AGAACAATGGGTCTAGTAGGTAGGCG -3'	
TLR8-F1	5'-GCATCATTTTGCCCCATAG -3'	PCR (F1+R1): Mutant 347bp PCR (F2+R2): Wild 344bp
TLR8-R1	5'-TGGTAGGCTGTCACTTTCTG -3'	
TLR8-F2	5'-GCATCATTTTGCCCCATAG -3'	
TLR8-R2	5'-TCGTCTGCTTTTCTGTTTCTC -3'	

Supplementary Table 2. A list of antibodies

Antibody	Supplier	Catalog No.	Application	Usage
Mouse β-actin antibody clone ARC5115-01	ABclonal	AC026	WB	1:5000
Rabbit-phospho-IKKα/β (S176/180) antibody	CST	2697	WB	1:2000
Rabbit IKKβ antibody	CST	8943	WB	1:2000
Rabbit phospho-NF-κB P65(S536) antibody	CST	3033	WB	1:500
Rabbit NF-κB P65 antibody	CST	3034	WB	1:1000
Rabbit phospho-TBK1 (S172) antibody	CST	5483S	WB	1:1000
Rabbit TBK1 antibody	Abcam	ab109735	WB	1:1000
Rabbit phospho-IRF3 (S379) antibody	CST	79945	WB	1:500
Rabbit IRF3 antibody	CST	4302	WB	1:1000
Rabbit RIG-I antibody	CST	4520	WB/IP	1:1000
Rabbit MDA5 antibody	CST	5321	WB/IP	1:1000
Rabbit VISA antibody	Santa Cruz	SC-365333	WB	1:1000
Mouse anti-Flag M2	Sigma-Aldrich	F1804	WB	1:2000
Goat anti-rabbit IgG, HRP	Pierce	31460	WB	1:2000
Mouse dsRNA antibody clone J2	Nordic Mubio	10010200	IF/IP	1:1000

Supplementary Table 3. A list of gRNA sequences

Primer Names	Sequence
Human RIG-I-gRNA	5’-GCTACATGGCCCCCTGGTTT-3’
Human MDA5-gRNA	5’-CTGGATGTACATTTTCACCC-3’
Human VISA-gRNA	5’-TACTTCATTGCGGCACTGAG-3’

Supplementary Table 4. A list of RT-qPCR primers

Primer Names	Sequence
EBIV-*S*- Forward	5’- GAAAAATGGCATCACCTGGGAAAGT -3’
EBIV-*S*-Reverse	5’-TTTTGGGTCCATCTCTTTCCTCTGC-3’
EBIV-*M*-Forward	5’-CAGGGCACTTACACAGCAGATGG-3’
EBIV-*M*-Reverse	5’-TCCCACACTTGTTCCACTCAATGC-3’
EBIV-*L*-Forward	5’-TGTCTGGGTTGCCATTAGTTGTAGC-3’
EBIV-*L*-Reverse	5’-ACTGCATGGGAGAGTTGACTTGATC-3’
GAPDH-Forward	5’-GACAAGCTTCCCGTTCTCAG-3’
GAPDH-Reverse	5’-GAGTCAACGGATTTGGTCGT-3’
IFNB1-Forward	5’-CATTACCTGAAGGCCAAGGA-3’
IFNB1-Reverse	5’-CAATTGTCCAGTCCCAGAGG-3’
CXCL10-Forward	5’-GGTGAGAAGAGATGTCTGAATCC-3’
CXCL10-Reverse	5’-GTCCATCCTTGGAAGCACTGCA-3’
TNF-Forward	5’-GCCGCATCGCCGTCTCCTAC-3’
TNF-Reverse	5’-CCTCAGCCCCCTCTGGGGTC-3’
MT-ND1-Forward	5’-CCCTAAAACCCGCCACATCT-3’
MT-ND1-Reverse	5’-GAGCGATGGTGAGAGCTAAG-3’
Gapdh-Forward	5’-GAAGGGCTCATGACCACAGT-3’
Gapdh-Reverse	5’-GGATGCAGGGATGATGTTCT-3’
Ifnb1-Forward	5’-TCCGAGCAGAGATCTTCAGGAA-3’
Ifnb1-Reverse	5’-TGCAACCACCACTCATTCTGAG -3’
Cxcl10-Forward	5’-GGTCTGAGTGGGACTCAAGG-3’
Cxcl10-Reverse	5’-GTGGCAATGATCTCAACACG-3’
Tnf-Forward	5’-GGTGATCGGTCCCCAAAGGGATGA-3’
Tnf-Reverse	5’-TGGTTTGCTACGACGTGGGCT-3’
Il6-Forward	5’-TCCATCCAGTTGCCTTCTTG-3’
Il6-Reverse	5’-GGTCTGTTGGGAGTGGTATC-3’
qPCR-EBIV-S-F（1）	5’-GTAGTGTACTCCACGCATAAAAC-3’
qPCR-EBIV-S-R（130）	5’-ACTCCCGATGTTAGTAGGG-3’
qPCR-EBIV-S-F（121）	5’-CATCGGGAGTACTTTTGACCC-3’
qPCR-EBIV-S-R（250）	5’-TTTTGCGAGACTATTTTTAATCTC-3’
qPCR-EBIV-S-F（241）	5’-TCTCGCAAAAAGAAGTGAATGGGAAG-3’
qPCR-EBIV-S-R（370）	5’-GATGCGGTGGAAGGTAAG-3’

qPCR-EBIV-S-F（361）	5’-CCACCGCATCAGCGGATA-3’
qPCR-EBIV-S-R（490）	5’-TTCCCAGGTGATGCCATTTTTCTC-3’
qPCR-EBIV-S-F（481）	5’-CACCTGGGAAAGTGGACCA-3’
qPCR-EBIV-S-R（610）	5’-TTTTGGGTCCATCTCTTTCCT-3’
qPCR-EBIV-S-F（601）	5’-GGACCCAAAATTCCTGG-3’
qPCR-EBIV-S-R（730）	5’-TTCTTCCAGCCAAGCCCTG-3’
qPCR-EBIV-S-F（721）	5’-CTGGAAGAAAACCAATGTC-3’
qPCR-EBIV-S-R（790）	5’-CCTTACATTCTGATTCCAAAC-3’
qPCR-EBIV-S-F（841）	5’-AAAGGGGTTTTTACCCCAAAC-3’
qPCR-EBIV-S-R（973）	5’-GTGTGCTCCACCTTAAAC-3’
qPCR-EBIV-M-F（601）	5’-ACATATGTCATGCGTTAGG-3’
qPCR-EBIV-M-R（701）	5’-TTAATGTGATGATTATAATGAG-3’
qPCR-EBIV-M-F（1601）	5’-GCACAAGAGCTGGTTACATC-3’
qPCR-EBIV-M-R（1701）	5’-TCTGTTATAGCACTCAGTG-3’
qPCR-EBIV-M-F（2601）	5’-GAGCCGATAAGTGGAAAG-3’
qPCR-EBIV-M-R（2701）	5’-GCAATATTGCCCTATGTCTGC-3’
qPCR-EBIV-M-F（3601）	5’-TTACACCTGTTATAGCGCCAG-3’
qPCR-EBIV-M-R（3701）	5’-GGAGATCCCCTTCCTGTTTTAG-3’
qPCR-EBIV-L-F（1601）	5’-TTAGCAGTCTCTCAATAC-3’
qPCR-EBIV-L-R（1701）	5’-GTTTTAATGTCTGAAGAAG-3’
qPCR-EBIV-L-F（2601）	5’-ATCTGAAAACAAAAGAATTT-3’
qPCR-EBIV-L-R（2701）	5’-AGCATCCTTATCATCAATGAAC-3’
qPCR-EBIV-L-F（3601）	5’-TGTGGCAAAACTATTTGAAGC-3’
qPCR-EBIV-L-R（3701）	5’-ACAGTGAGACAAACTC-3’
qPCR-EBIV-L-F（4601）	5’-ATTGGTAGAGTCACCTACCC-3’
qPCR-EBIV-L-R（4701）	5’-CAATACTTGAATACGATC-3’
qPCR-EBIV-L-F（5601）	5’-GAGAAGATAATAAATTGAAAATTGC-3’
qPCR-EBIV-L-R（5701）	5’-ATTATGCCTAGAGTTCAAC-3’
qPCR-EBIV-L-F（6601）	5’-TTTGCCAGCAGTAAAGAAAGAG-3’
qPCR-EBIV-L-R（6701）	5’-ATTATGCCTAGAGTTCAAC-3’

Supplementary Table 5. A list of PCR primers

Primer Names	Sequence
PCR-EBIV-S1-F（120）	5’-ACATCGGGAGTACTTTTG-3’
PCR-EBIV-S1-R（680）	5’-CAAGCTTAGTTGTTGTCC-3’
PCR-EBIV-S2-F（290）	5’-AAGGTGCCTGTACTCAATACG-3’
PCR-EBIV-S2-R（850）	5’-AACCCCTTTTTGGTCGAA-3’
PCR-EBIV-M1-F（53）	5’-ATGGCGATTTCTATTGTCTTG-3’
PCR-EBIV-M1-R（1100）	5’-CATTTAGTGTATACTTCCTATC-3’
PCR-EBIV-M2-F（1101）	5’-AGGTGAATGATATAGTCCTTGGG-3’
PCR-EBIV-M2-R（2200）	5’-AGCAATCCATTCTTGCTTGAGC-3’
PCR-EBIV-M3-F（2201）	5’-AAATCGCTAAGCTTAGTGAGG-3’
PCR-EBIV-M3-R（3300）	5’-ATTACATCTTGACAGGAACC-3’
PCR-EBIV-M4-F（3301）	5’-CAGAACAGAAACAAAGGTTT-3’
PCR-EBIV-M4-R（4360）	5’-TTATCTTTTCTTCATCTCCATC-3’
PCR-EBIV-L1-F（47）	5’-ATGGAGGATCCAATGTATGAGC-3’
PCR-EBIV-L1-R（1100）	5’-CCATACTAACACCTATAGCC-3’
PCR-EBIV-L2-F（1101）	5’-ATATTGGTAATAATATTGGATTG-3’
PCR-EBIV-L2-R（2200）	5’-CGTTATATCATAATCTGATAGAT-3’
PCR-EBIV-L3-F（2201）	5’-CAAAAAGGGATAAATGAC-3’
PCR-EBIV-L3-R（3300）	5’-TCGTCCGGAAGGATCAAATTTTTC-3’
PCR-EBIV-L4-F（3301）	5’-CTTATTATCCAATATAATGGAT-3’
PCR-EBIV-L4-R（4400）	5’-TTTCGCCTTTTGTGACAAGAAG-3’
PCR-EBIV-L5-F（4401）	5’-ATAAAGAACAATATATAAACTCC-3’
PCR-EBIV-L5-R（5500）	5’-GTTTGTTTTCAATGCATC-3’
PCR-EBIV-L6-F（5501）	5’-GAAAGAGTATCATGGAATAAC-3’
PCR-EBIV-L6-R（6763）	5’-TCAGAAGAATGTGAACATAGAC-3’
pET-28a-Vec-F	5’-CATCATCATCATCATCACAGC-3’
pET-28a-Vec-R	5’-GGTATATCTCCTTCTTAAAG-3’
pET-28a-EBIV-NP-F	5’-CTTTAAGAAGGAGATATACC ATGTTGGAGCTAGAATT-3’
pET-28a-EBIV-NP-R	5’-GATGATGATGATGATGCATT CTGATTCCAAAC-3’
